# Reprogramming tumor-associated macrophages with lipid nanosystems reduces PDAC tumor burden and liver metastasis

**DOI:** 10.1186/s12951-024-03010-5

**Published:** 2024-12-24

**Authors:** Adrián Palencia-Campos, Laura Ruiz-Cañas, Marcelina Abal-Sanisidro, Juan Carlos López-Gil, Sandra Batres-Ramos, Sofia Mendes Saraiva, Balbino Yagüe, Diego Navarro, Sonia Alcalá, Juan A. Rubiolo, Nadège Bidan, Laura Sánchez, Simona Mura, Patrick C. Hermann, María de la Fuente, Bruno Sainz

**Affiliations:** 1 Cancer Stem Cells and Fibroinflammatory Microenvironment Group, Instituto de Investigaciones Biomédicas (IIBm) Sols-Morreale CSIC-UAM, 28029 Madrid, Spain; 2 Biomarkers and Personalized Approach to Cancer Group (BIOPAC), Instituto Ramón y Cajal de Investigación Sanitaria (IRYCIS), Area 3 Cancer, 28049 Madrid, Spain; 3 Biobanco Hospital Universitario Ramón y Cajal, IRYCIS, Madrid, Spain; 4 Nano-Oncology and Translational Therapeutics Group, IDIS, Complexo Hospitalario Universitario de Santiago de Compostela, 15706 Santiago de Compostela, Spain; 5 University of Santiago de Compostela (USC), 15782 Santiago de Compostela, Spain; 6 Centro de Investigación Biomédica en Red, CIBERONC, ISCIII, Área Cáncer, Madrid, Spain; 7 Department of Biochemistry, Autónoma University of Madrid (UAM), 28029 Madrid, Spain; 8 Department of Zoology, Genetics and Physical Anthropology, Faculty of Veterinary, University of Santiago de Compostela (USC), Lugo, Spain; 9 Laboratorio Mixto de Biotecnología Acuática, Facultad de Ciencias Bioquímicas y Farmacéuticas, UNR, 2000 Rosario, Argentina; 10 Université Paris-Saclay, CNRS, Institut Galien Paris-Saclay, 91400 Orsay, France; 11 Department of Internal Medicine I, Ulm University, Ulm, Germany; 12 DIVERSA Technologies S.L, Edificio Emprendia, Campus Sur, 15782 Santiago de Compostela, Spain

**Keywords:** Pancreatic ductal adenocarcinoma, Tumor-associated macrophages, Tumor microenvironment, Lipid nanoemulsions, Galunisertib, Liver metastasis, Reprogramming

## Abstract

**Background:**

Pancreatic ductal adenocarcinoma (PDAC) requires innovative therapeutic strategies to counteract its progression and metastatic potential. Since the majority of patients are diagnosed with advanced metastatic disease, treatment strategies targeting not only the primary tumor but also metastatic lesions are needed. Tumor-Associated Macrophages (TAMs) have emerged as central players, significantly influencing PDAC progression and metastasis. Our objective was to validate an innovative therapeutic strategy involving the reprogramming of TAMs using lipid nanosystems to prevent the formation of a pro-metastatic microenvironment in the liver.

**Results:**

In vitro results demonstrate that M2-polarized macrophages lose their M2-phenotype following treatment with lipid nanoemulsions composed of vitamin E and sphingomyelin (VitE:SM), transitioning to an M0/M1 state. Specifically, VitE:SM nanoemulsion treatment decreased the expression of macrophage M2 markers such as *Arg1* and *Egr2*, while M1 markers such as *Cd86*, *Il-1b* and *Il-12b* increased. Additionally, the TGF-βR1 inhibitor Galunisertib (LY2157299) was loaded into VitE:SM nanoemulsions and delivered to C57BL/6 mice orthotopically injected with KPC PDAC tumor cells. Treated mice showed diminished primary tumor growth and reduced TAM infiltration in the liver. Moreover, we observed a decrease in liver metastasis with the nanoemulsion treatment in an intrasplenic model of PDAC liver metastasis. Finally, we validated the translatability of our VitE:SM nanosystem therapy in a human cell-based 3D co-culture model in vivo, underscoring the pivotal role of macrophages in the nanosystem’s therapeutic effect in the context of human PDAC metastasis.

**Conclusions:**

The demonstrated effectiveness and safety of our nanosystem therapy highlights a promising therapeutic approach for PDAC, showcasing its potential in reprogramming TAMs and mitigating the occurrence of liver metastasis.

**Graphical abstract:**

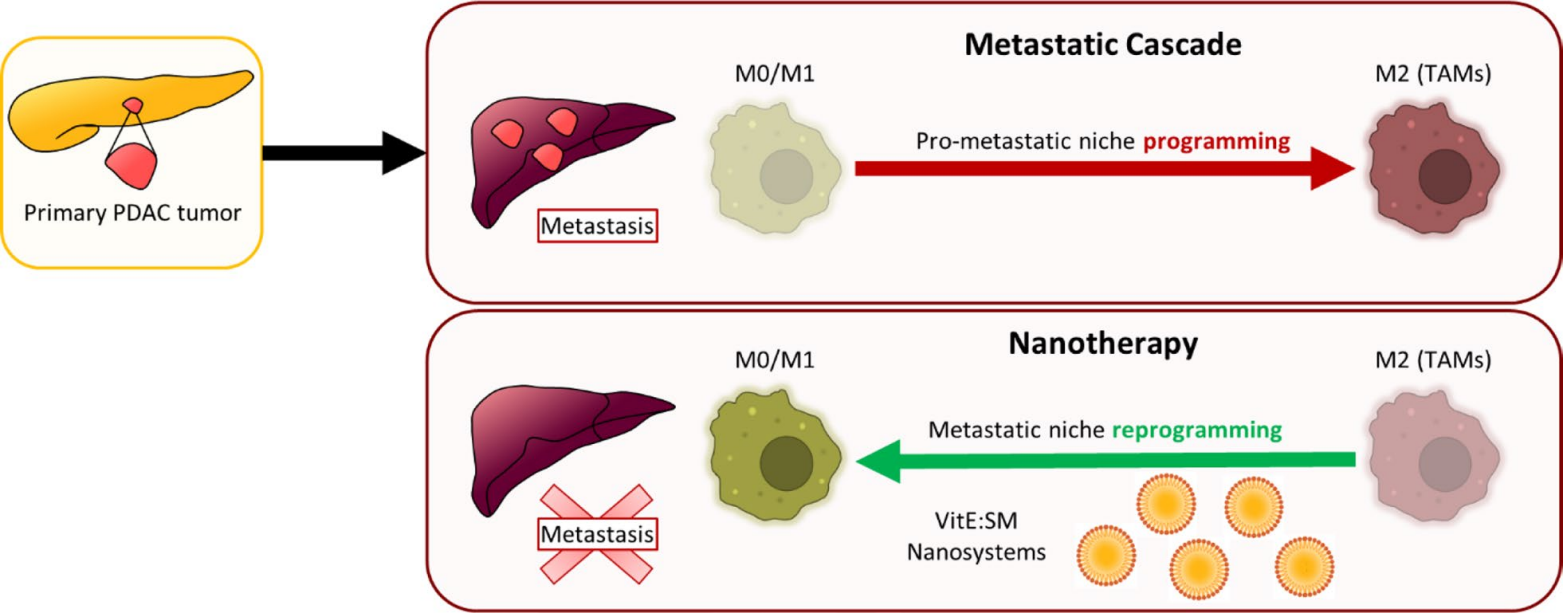

**Supplementary Information:**

The online version contains supplementary material available at 10.1186/s12951-024-03010-5.

## Background

Pancreatic ductal adenocarcinoma (PDAC) is the fourth leading cause of cancer-related deaths in the United States [1], a consequence of various factors such as delayed diagnosis, the absence of specific markers and symptoms of early detection and the inherent metastatic nature of this tumor [2, 3]. Notably, when PDAC is identified at an early stage, the 5-year overall survival rate following surgical resection of the primary tumor reaches 44% [4]; however, fewer than 20% of cases are eligible for surgical intervention [5–7]. Even in cases where the primary tumor is successfully resected, approximately 75% of patients develop metastatic relapse within five years post-resection [8–10].

The metastatic process of PDAC occurs through the bloodstream [11], with the liver being the primary target. Liver metastases are detected in 76–80% [12] of patients, limiting their eligibility for surgery [13, 14]. Additionally, metastases can also occur in the peritoneum (48%) and the lungs (45%), further complicating the management of PDAC patients [12]. Since a substantial proportion of patients present with metastases at the time of diagnosis, it is imperative that we develop treatment strategies that target not only the primary tumor but also metastatic lesions.

Metastatic dissemination in PDAC occurs through a series of orchestrated events [15]. PDAC cells release exosomes containing factors that are taken up by liver Kupffer cells, stimulating the production of fibrotic cytokines such as transforming growth factor-beta (TGF-β) [15]. TGF-β plays a crucial role in metastasis formation, participating in epithelial-mesenchymal transition (EMT), activation of cancer-associated fibroblasts (CAFs), and immune suppression [16, 17]. These cytokines stimulate hepatic stellate cells, prompting the synthesis and deposition of fibronectin, which establishes a fibrotic microenvironment in the liver. This environment promotes the recruitment of bone marrow-derived cells, including macrophages and neutrophils [18, 19]. These sequential processes culminate in the formation of a pre-metastatic niche [20], creating favorable conditions for the survival, proliferation, and subsequent metastatic growth of disseminated PDAC cells in the liver [15].

Within this dynamic metastatic tumor microenvironment, CAFs and immune cells, including macrophages, T cells, and neutrophils, create a niche for cancer cells to modulate tumor growth and their invasive behavior [21–23]. Tumor-associated macrophages (TAMs), in particular, have emerged as central orchestrators of PDAC tumorigenesis, significantly impacting the progression and metastasis of PDAC. Through interactions with PDAC cells, TAMs influence disease progression by facilitating resistance to chemotherapeutics, promoting metastasis and influencing cancer cell evasion of the antitumor immune response [24–26]. Macrophages, recognized for their plasticity, are broadly classified into M1 and M2 subtypes. M1 macrophages, characterized by high expression of CD86, IL-1β, and IL-12β, exhibit a pro-inflammatory profile and suppress tumor growth and progression [27]. On the other hand, M2 macrophages, characterized by high expression of CD206, ARG1, and CD163, promote tumor progression by antagonizing anti-tumor T-cell immunity [28]. TAMs often express an M2-like phenotype, which is associated with tumor-promoting activities [29]. The collaboration between PDAC cells and TAMs significantly contributes to disease progression, making TAMs an attractive target for alternative therapies [30, 31]. By shifting the polarization state of TAMs from the tumor-promoting M2 phenotype to the anti-tumoral M1 phenotype, one could potentially alter the tumor microenvironment, creating a hostile setting for cancer progression and preventing PDAC metastasis.

Nanotechnology is emerging as a promising tool in the biomedical field, with recent advancements focusing on reprogramming TAMs using nanomaterials, offering new perspectives for designing nanotechnology-based therapies to enhance TAM-mediated immunotherapy [32, 33]. Specifically, organic nanoparticles are particularly attractive due to their translational potential, which can be attributed to their biocompatibility and biodegradability. Along these lines, nanoemulsions composed of vitamin E (VitE) and the biocompatible lipid sphingomyelin (SM) stand out as exceptionally promising carriers for delivering innovative therapeutics and advancing novel biomedical applications [34], a trend observed across various cancer types [35–37]. These lipid nanosystems are readily phagocytosed by macrophages and can easily encapsulate therapeutics, such as potential macrophage reprogramming agents. Thus, they represent highly effective vehicles for targeted delivery to, and reprograming of, TAMs. They have also proved to accumulate in the liver, being promising agents for treating liver metastasis [38, 39]. In terms of composition, VitE has been extensively utilized in numerous formulations intended for clinical use, due to its good biodegradability and versatility in associating and encapsulating a variety of therapeutic molecules. It is also a potent antioxidant and recent discoveries highlight that VitE also has direct impacts on the immune system, particularly on macrophages [40–43]. On the other hand, sphingolipids present a common structural feature, the sphingosine backbone, which has bioactive functions, from cell growth regulation to modulators of cell-to-cell interactions [44, 45]. Specifically, SM is known to be a major component in lipid bilayers and serves as a precursor for ceramide, a bioactive lipid molecule involved in cell signaling pathways that regulates apoptosis, proliferation, and inflammation. The role of SM in modulating macrophage activation and resulting inflammation has also been described [46, 47].

The objective of this study was to test an innovative nano-therapeutic approach involving reprogramming TAMs to an M0/M1 state using a battery of lipid nanosystems, including VitE:SM nanoemulsions as a means of inhibiting the formation of the pro-tumorigenic liver microenvironment that facilitates PDAC metastasis. This targeted reprogramming holds promise not only in restraining the influence of TAMs on primary tumor growth but also in mitigating their pro-metastatic impact in distant organs, particularly the liver. In addition, our proposed approach capitalizes on the potential of these specific types of lipid nanosystems for the precise and effective delivery of therapeutic agents already employed in the clinical settings to target the pre-metastatic niche, such as the TGF-βR1 inhibitor Galunisertib [48–50]. This strategic and clinically-relevant avenue of research offers a promising method for combating PDAC metastasis, reshaping the landscape of pancreatic cancer treatment and offering hope and targeted solutions for patients.

## Methods

**Fig. 1:**
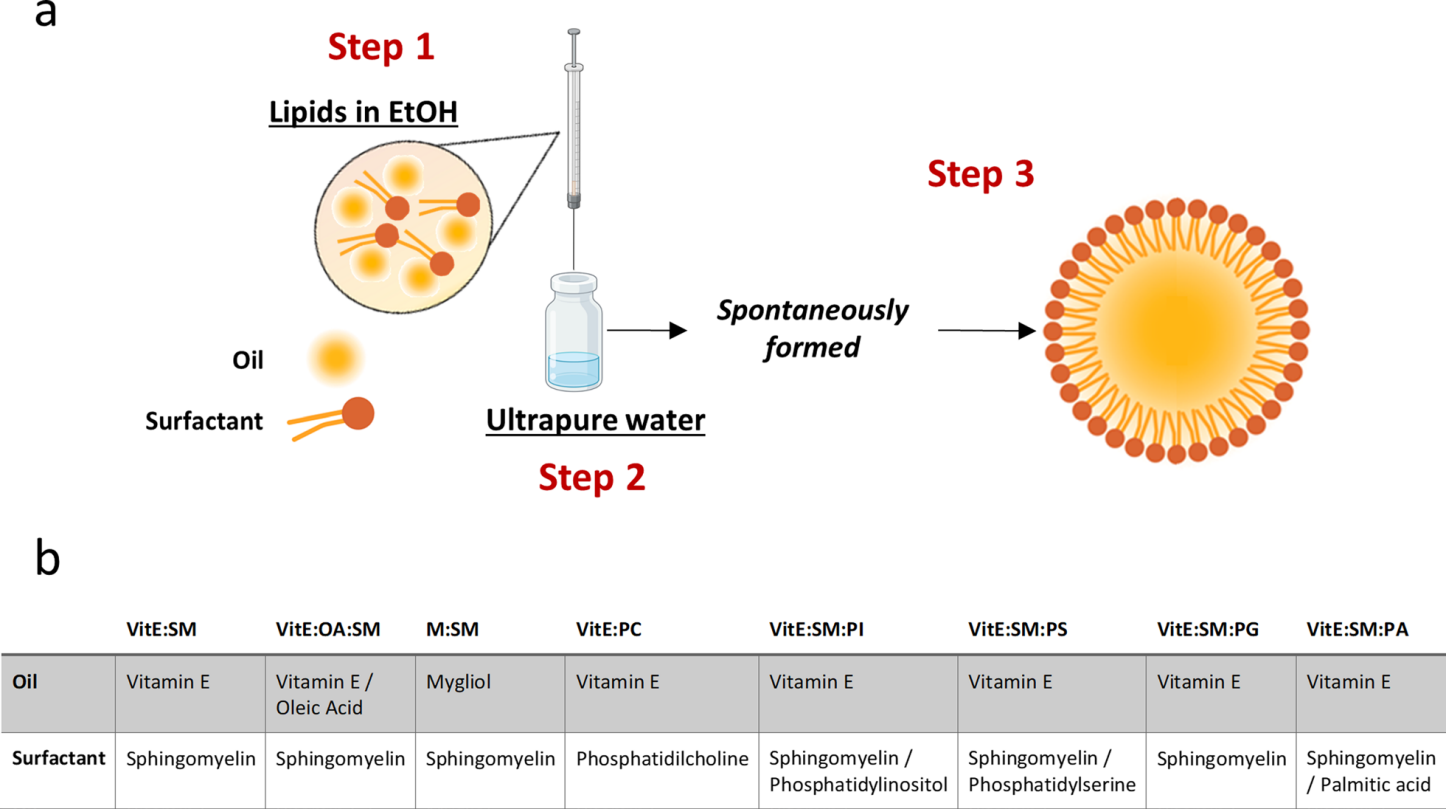
Development and characterization of nanosystems. **a** Schematic representation depicting the process of generating nanoemulsions through the ethanol injection method. Briefly, oils (VitE and/or OA and M) and surfactants (SM and/or PC, PA, PI, PS and PG) were mixed in an ethanolic organic phase (step 1). Subsequently, this mixture was injected in ultrapure water (step 2) and the diverse nanosystems were spontaneously formed (step 3) at room temperature. **b** Summary of the components that make up the lipid nanosystems. *VitE* Vitamin E, *SM* Sphingomyelin, *OA* Oleic acid, *M* Mygliol, *PC* Phosphatidylcholine, *PI* Phosphatidylinositol, *PS* Phosphatidylserine, *PG* Phosphatidylglycerol, *PA* Palmitic acid

**Lipid nanosystems preparation.** Nanosystems comprising VitE, SM, OA, M, PA, PI, PS, and PG lipids were formulated by adapting the straightforward and highly efficient ethanol injection method previously outlined by our group [35, 37] (Fig. 1a). In short, various lipid compositions (Fig. 1b, Table 1) were dissolved in 100 μL of 96% EtOH. Subsequently, nanosystems were promptly generated by injecting the organic phase into 1 mL of ultrapure water at room temperature (RT). The nanosystems were stored at 4°C until further use. Formulation purification and concentration were achieved using spin filters (Amicon® Ultra Centrifugal Filters, 100K, 0.5mL, Merck) in a refrigerated microcentrifuge (5417R; Eppendorf). The spin filters were initially rinsed with ultrapure water at 6000×g for 5 min. The formulations were then transferred to the spin filters and centrifuged for 5 min at 4°C and 3000×g. Four additional washes were performed with ultrapure water, following the same centrifugation parameters. Finally, the formulations were collected for further experimentation.

**Table 1:** Physicochemical properties of lipid nanosystems measured, after preparation, by dynamic light scattering (DLS) and laser doppler anemometry (LDA) including hydrodynamic size, polydispersity index (PdI) and zeta potential (ZP)

Formulation	Mass ratio	Size (nm)	PDI	ZP (mV)
VitE:SM	1:0.1	97 ± 5	0.08 ± 0.02	– 13 ± 8
VitE:OA:SM	0.5:0.1:0.5	101 ± 3	0.21 ± 0.02	– 27 ± 1
M:SM	1:0.1	93 ± 6	0.10 ± 0.05	–10 ± 4
VitE:PC	1:0.1	125 ± 6	0.04 ± 0.01	– 5 ± 2
VitE:SM:PI	1:0.1:0.002	66 ± 3	0.20 ± 0.06	– 4 ± 2
VitE:SM:PS	1:0.1:0.002	91 ± 7	0.22 ± 0.04	– 7 ± 2
VitE:SM:PG	1:0.1:0.002	97 ± 4	0.10 ± 0.02	– 12 ± 3
VitE:SM:PA	1:0.1:0.05	100 ± 8	0.19 ± 0.03	– 12 ± 1
VitE:SM:PA002	1:0.1:0.002	95 ± 2	0.09 ± 0.02	– 10 ± 4

Results are expressed as mean ± SD, n = 5. *nm*: nanometer, *PDI* polydispersity index, *ZP* zeta potential in millivolts (mV).

**Loading of LY2157299.** LY2157299 (Cat No. HY-13226, MedChem), previously dissolved in DMSO, was added to the lipids dissolved in 100 μL of 96% EtOH, at a theoretical loading of 10% (w/w with respect to the total lipid content), prior to injection in the aqueous phase. VitE:SM nanoemulsions loaded with LY2157299 (VitE:SM:LY) were obtained. HPLC analyses, performed in an HPLC 1260 Infinity II (Agilent, USA) system equipped with a G7111A pump, a G7129A autosampler, and a G7114A UV–Vis detector set at 236nm, and with a LUNA C18 column (5 µm, 100Å pore size, 4.6 mm × 150 mm) (Phenomenex Inc., US) operating at 25 ºC, was used for quantification of the encapsulated drug. The mobile phases used were solvent A (0.1% acetic acid, constant), solvent B (50% methanol), and solvent C (50% acetonitrile). The flow rate was set at 1 mL/min. Separation was achieved by mobile phase; drug peak (LY) at minute 2, VitE/SM peak at minutes 11–12. Total run 14 min.

**Preparation of TF labelled VitE:SM nanoemulsions.** Fluorescent labelled VitE:SM nanoemulsions were formulated as previously described in the above sections “Lipid nanosystems preparation” and “Loading of LY2157299”. Briefly, sphingomyelin-TopFluor® (4.5 μg) (Cat No. 810265, Avanti Polar Lipids) and/or LY2157299 (0.55 mg) were combined with other organic phase compounds (VitE and SM) in a final volume of 100 μL of 96% EtOH. The resulting organic phase was swiftly injected into 1 mL of ultrapure water, immediately forming the nanosystems at RT.

**Physicochemical characterization of the lipid nanosystems.** Particle size and polydispersity index (PDI) were determined by Dynamic Light Scattering (DLS) and Z-potential values by Laser Doppler Anemometry (LDA), using a Zetasizer NanoZS® (Malvern Instruments, Worcestershire, UK). Samples were diluted 1:10 with ultrapure water and the measurements were performed at 25°C with a detection angle of 173°. Subsequently, all developed formulations were evaluated in relevant biological media to ensure the maintenance of their properties during in vitro assays. Formulations were incubated at 37°C with 1% FBS (Fetal Bovine Serum) supplemented RPMI 1640 (Corning) at a 1:6 (v/v) dilution under orbital shaking (300 rpm). Additionally, VitE:SM mean particle size and morphology distribution were observed by Field Emission Scanning Electron Microscopy (FESEM) using a ZEISS FESEM ULTRA Plus microscope (Carl Zeiss Micro Imaging, GmbH, Germany) configured with InLens and STEM modes and operating at 20 kV. Twenty µL (0.50 mg/mL) of filtered nanosystems were stained with phosphotungstic acid (2% w/v) at a ratio 1:1. Next, 10 µL were placed on a carbon coated grid and left for 30s. The grid was allowed to dry and then, repeatedly washed with filtered ultrapure water. Finally, it was left dry until its visualization.

**Cell culture and reagents.** All murine (mu) and human (hu) cells utilized in the study (muIBMDM, muBMDM, muKPC, huPANC1, huCAFs, and hu macrophages) were cultured in RPMI 1640 1X (Cat no. 15–040-CV, Corning) supplemented with 10% FBS (Gibco), 50 units/ml penicillin/streptomycin (Cat no. 15–040-CV, Gibco), L-Glutamine (Cat no. BE17-605E, Lonza) and Amphotericin B (Cat no. 15290–018, Gibco) at 37°C with 5% CO_2_. MuIBMDM cells were obtained from Dr. Antonio Castrillo's laboratory (IIBM), and hu macrophages were obtained from excess buffy coat-derived PBMCs from Dr. Lisardo Boscá's laboratory (IIBM). Primary muBMDM cells were extracted from the bone marrow of the femur and tibia of euthanized C57BL/6 mice and cultured with 10 ng/mL of murine M-CSF (Cat no. 315–02, PeproTech). The mu PDAC KPC cell line was derived from a spontaneous tumor originating in a genetically engineered LSL-Kras^G12D/+^; LSL-Trp53^R172H/+^; Pdx-1-Cre (KPC) C57Bl/6 mouse model. Primary huCAFs were established via an outgrowth method from a freshly digested primary human resectable PDAC tumor as already described [51]. When fluorescence-marked cells were required, KPC cells and CAFs were infected with an mCherry expressing lentivirus (PRRL CMV IRES mCherry) as described in [52], and PANC1 cells were fluorescently-labeled with a GFP expressing lentivirus (PRRL CMV GFP). Regular mycoplasma testing was conducted on all cells. For macrophage polarization, primary muBMDM or muIBMDM cells were cultured for 24 h with 10 ng/ml of IL-4 (Cat no. 214–14, PeproTech) for M2 or 10 ng/ml of LPS (Cat. no L4391, Sigma) + IFN-γ (Cat. no 315–05, Peprotech) for M1. MuIBMDM cells were seeded at a density of 4 × 10^5^ cells/p60 prior to Western blot and qRT-PCR analyses. For cell culture treatments with nanoemulsions, all tested compositions were used at 1 mg/mL for 4 h. In the experiment comparing VitE:SM with VitE:SM:LY, nanoemulsions were used at two concentrations, 0.5 mg/mL and 1 mg/mL, for 4 h. In the experiment evaluating the synergy between VitE and SM, VitE:SM nanoemulsions were used at 1 mg/mL, SM at 0.1 mg/mL, VitE at 0.9 mg/mL and the free combination of VitE and SM at 0.9 mg/mL and 0.1 mg/mL, respectively, all for 4 h. In the remaining experiments, nanoemulsion concentrations are indicated in the figure legends.

**Cellular toxicity assay.** The toxicity of nanoemulsions on macrophages was assessed using the Toxilight BioAssay kit (Lonza, Walkersville, MD) in accordance with the manufacturer's instructions.

**RNA preparation and real‑time PCR.** RNA from macrophage cultures and tissues was extracted using the GTC method following the protocol described in [53]. For cDNA synthesis, 1 µg of isolated RNA was retrotranscribed using the Thermo Scientific Maxima First Strand cDNA Synthesis Kit (Cat. no K1671, ThermoFisher Scientific), following the manufacturer's instructions. SYBR green-based qRT-PCR (Cat. no A25742, PowerUp™ SYBR™ Green Master Mix, ThermoFisher Scientific) was performed using an Applied Biosystems StepOnePlus™ real-time thermocycler (ThermoFisher Scientific). The PCR conditions included an initial 10-min denaturation step at 95°C, followed by 40 cycles of denaturation (15 s at 95°C) and annealing/extension (1 min at 60°C). mRNA copy numbers were determined relative to standard curves composed of serial dilutions of plasmids containing the target coding sequences and normalized to *Actb* levels. The primers used in this study are listed in Table S1.

**RNA sequencing analysis.** RNA was extracted from muBMDM macrophages polarized to M2 with IL4 and treated or untreated with VitE:SM nanoemulsions (1 mg/mL), following the procedures outlined in the previous methods section. Genes with a fold change > 1 and a p-value < 0.05 were considered differentially upregulated and genes with a fold change < −1 and a p-value < 0.05 were considered differentially downregulated after the comparison of gene expression in both M2 and VitE:SM samples. Genes were ranked according to LOG2 fold change and Gene Set Enrichment Analysis was performed employing GSEA v4.3.2. (Broad Institute). Gene signatures were obtained from Molecular Signature Database (MsigDB) and from signatures published by other groups. Signatures with a NOM p-value < 0.05 and an FDR q-value < 0.05 were considered significantly enriched. Volcano plots and GSEA histograms were made employing Graph Pad Prism 8, and GSEA plots were obtained with GSEA v4.3.2. FASTQ data files generated have been deposited in Sequence Read Archive (SRA) of the National Center for Biotechnology Information (NCBI) with accession number ID: PRJNA1099468.

**Western blot.** Cells were lysed in RIPA Buffer (Cat. no R0278-50mL, Sigma-Aldrich), supplemented with a protease inhibitor cocktail (Roche Applied Science, Indianapolis, IN). The extracts were clarified for 15 min at 13,300 rpm at 4°C, and protein concentrations were determined using the BCA Protein Assay Kit (Cat no. 23227, Pierce). For Western blot analysis, 60 µg of clarified protein was separated on 10% SDS–PAGE gels and transferred to 0.45 µM PVDF membranes (Cat no. IPVH00010, Merck Millipore). For protein denaturalization, NuPAGE (Cat no. NP0007, Invitrogen) and 2-Mercaptoethanol (Cat no. M7522, Sigma) were used. Membranes were blocked with PBS-T (PBS 1X + 1% Tween20 (v/v) (Cat no. BP337-5000, Fisher bioreagents)) + 5% BSA (w/v) (Cat no. MD04602, Nzytech) and incubated with anti-mouse ARG1 (1:1000, Cat no. sc-271430, Santa Cruz Biotechnology) or anti-mouse beta Tubulin (1:2000, Cat no. E-AB-20033, Elabscience) overnight at 4°C. Three washes with PBS-T were performed before and after incubation with secondary horseradish peroxidase-conjugated goat anti-rabbit or goat anti-mouse antibodies (Amersham) for 1 h. Western blots were developed using SuperSignal chemiluminescent substrate (Cat no. 34577, Thermo Scientific), and images were captured using an iBright™ 1500 Imaging System (Invitrogen). Densitometric analysis was conducted using ImageJ, and indicated molecular weights were obtained from a PageRuler™ Plus Prestained protein ladder (Cat no. 26619, Thermo Scientific).

**Formalin-fixed paraffin-embedded (FFPE) tissue dissociation.** Tissues embedded in paraffin were sectioned at 50 microns using a microtome, and a single-cell suspension was prepared with the MACS FFPE Tissue Dissociation Kit (Miltenyi, Cat. No 130–118-052). Briefly, tissue sections were deparaffinized, then dissociated chemically, enzymatically, and mechanically, followed by washing and filtration. After obtaining the single-cell suspension, the cells were blocked and stained for flow cytometry.

**Tissue digestion and cell isolation.** Tissues extracted from mice were minced and enzymatically digested with Collagenase P (Cat no. 11213857001, Roche) and Dispase (Cat no. 17105–041, Gibco) for 30 min at 37°C, vortexing every 10 min. Samples were passed through a 40µM Fisherbrand™ Sterile Cell Strainer (Cat no. 11587522, FisherScientific) and cells were collected in a new tube, where they were centrifugated at 1,300 rpm for 5 min. After a 1X PBS wash, cells were ready for flow cytometric analysis as described below.

**Flow cytometry.** Mouse cell cultures or cells extracted from tissues or FFPE blocks were blocked with purified anti-mouse CD16/CD32 (Fc Shield) (1:100, Cat no. 70-016-U500, TONBO) for 10 min at 4°C and then washed with 1X PBS prior to cell surface marker incubation for 30 min at 4°C with the following antibodies: CD45 (PE-Cyanine7), CD11b (PerCP-Cyanine5.5), F4/80 (APC) and CD206 (PE) or CD45 (PE-Cyanine7)/CD206 (PE-Cyanine7), CD11b (PerCP-Cyanine5.5) and F4/80 (APC) for the intrasplenic experiment (Table S2). Human macrophages were blocked with Flebogamma (Grifols) for 15 min at 4°C and then resuspended in Flow buffer [1X PBS; 3% FBS (v/v); 3 mM EDTA (v/v)] before analysis. For detection of M2 macrophage markers on control and nanoemulsion-treated human M2-polarized macrophage cultures, the mouse monoclonal anti-human-CD163-PE antibody and the mouse monoclonal anti-human-CD206-FITC antibody were used (Table S2). Prior to analysis, all samples were washed with 1X PBS and cells were analyzed with a 4-laser Attune NxT Acoustic Cytometer (Thermo Fisher Scientific). To exclude dead cells, samples were resuspended in Flow buffer [1X PBS; 3 mM EDTA (v/v)] with 2 µg/mL of 4′,6-diamidino-2-phenylindole (DAPI) before analysis. Results were analyzed with FlowJo software (Tree Star Inc., Ashland, OR.).

**Microscopy images.** For nanoparticle visualization, muBMDM cells were seeded in Glass Bottom Dish 35 mm plates (Cat no. P35G-1.5–14-C, Mattek) and treated for 24h with VitE:SM:TF nanoemulsions (0.5 mg/mL). Cells were washed with 1X PBS prior to Hoechst 33342 (Cat no. 62249) staining of nuclei. Images were acquired with a laser scanning confocal microscope Zeiss 710 40X Apochromatic and were analyzed using the softwares ZEN 2009 and ImageJ. Culture plate images were acquired with an Invitrogen™ EVOS™ Digital Color Fluorescence Microscope (Cat no. 12–563-340, Thermo Fisher Scientific).

**Histology.** For histopathological analysis, a section of each organ was fixed in 10% buffered formalin and paraffin embedded. FFPE blocks were serially sectioned (3 μm thick) with a microtome LEICA RM 2125 RTS and stained with hematoxylin and eosin (H&E). The stained sections were covered with DPX mounting medium fast (Cat no. 255254.1610, Panreac AppliChem) and a coverslip. Images were acquired with a Motic EasyScan One slide scanner and analyzed with Motic DS Assistant software.

**DNA extraction and PCR.** DNA from tissues were extracted with the QIAcube Connect equipment (Cat no. 9002864, QIAGEN), TissueLyser LT (Cat no. 85600, QIAGEN) and DNeasy Blood & Tissue kit (Cat no. 51106, QIAGEN) following the manufacturer´s instructions. PCR amplification was performed with DreamTaq Green PCR Master Mix (2X) (Cat no. K1081, Thermo Scientific) and a SimpliAmp™ (Cat no. A24812, ThermoFisher Scientific) thermocycler. The amplification protocol was as follows: 5 min denaturation step at 95°C, 35 cycles of denaturation (30 s at 95°C), annealing (30 s at 67°C) and extension (45 s at 72°C), and a final extension of 5 min at 72°C. PCR products were resolved in an agarose (1%) gel (Cat no. 8019.00, Condalab) with SybrSafe (Cat no. S33102, Invitrogen) staining for 45 min at 100 V, and images were acquired with an iBright™ 1500 Imaging System (Invitrogen). As a reference for molecular weight, the 1kb Plus DNA Ladder (Cat no. 10787018, Invitrogen) was used. Primers used for CRE and murine *Gapdh* amplification have the following sequences: CRE: forward: 5´-gcctgcattaccggtcgatgcaacga-3´; reverse: 5´-gtggcagatggcgcggcaacaccatt-3´, *Gapdh* forward: 5´-gcctgcattaccggtcgatgcaacga-3´; reverse: 5´-gtggcagatggcgcggcaacaccatt-3´.

**Magnetic resonance imaging (MRI).** Anesthetized animals (induced with 2–2.5% and maintained with 1–1.5% isoflurane/oxygen) were placed in a supine position in a methyl methacrylate holder in the MRI system, and restrained with a bite-bar attached to a nose mask and a small piece of tape over the head and attached to the animal’s bedding. A heated probe was employed to maintain the core body temperature at approximately 37°C. Physiological status of the animals was monitored by a gating system designed for small animals using respiratory rate and body temperature. Image acquisitions were carried out in a Bruker BioSpec® 7 T system (Bruker Biospin, Ettlingen, DE), 16 cm bore, with a 90 mm gradient insert of 360 mT/m and a 40 mm quadrature volume resonator, interfaced with an Avance III radiofrequency console and running ParaVision 6.0.1 software. Anatomical T2-weighted images were acquired in axial coronal orientation for tumor assessment: TR (repetition time): 1200 ms; TE (echo time): 30.0 ms; FA (flip angle): 180°; FoV (field of view): 40 mm × 40 mm; matrix: 264 × 264; 24 slices of 0,8 mm per slice, acquisition time: 15 min per orientation.

**In vivo orthotopic experiments.** Mice were housed according to previously reported protocols [54]. All in vivo procedures in mice were conducted in accordance with protocols approved by the local Animal Experimental Ethics Committee of the Instituto de Salud Carlos III (PA 34–2012) or the Use Committee for Animal Care from the Universidad Autónoma de Madrid (UAM) (Ref# CEI-25–587) and the Comunidad de Madrid (PROEX 335/14 or 294/19). C57BL/6 mice (Charles River Co), aged 6–9 weeks, underwent orthotopic pancreatic implantation with KPC cells (2,500 cells/pancreas) resuspended in 50 µL of a 3:2 ratio of Matrigel (Cat no. 354234, Corning):RPMI. Treatments commenced 4–6 days post-implantation, with nanoemulsion doses of 69 mg/kg administered intraperitoneally and 25 mg/kg administered retro-orbitally or intravenous tail vein. For nanoemulsion concentration or buffer exchange, Centrifugal Filters Amicon Ultra—0.5mL Ultracel-100K (Cat no. UFC510096, Merck Millipore) were employed following the manufacturer's instructions. Three weeks after treatment initiation, mice were euthanized and tumors and livers were weighed and photographed after animal perfusion with cold 1X PBS.

**Liver function test.** Blood samples were taken by cardiac puncture. EDTA tubes were centrifuged and plasma was collected. Determination of liver enzymes were performed by a clinically-certified laboratory (Laboratorios Echevarne, Spain) and compared to normal references values (Charles River Co., https://www.criver.com/sites/default/files/resources/doc_a/C57BL6MouseClinicalPathologyData.pdf.)

**Intrasplenic and intravenous model.** C57BL/6 mice (Charles River Co), aged 6–9 weeks, underwent intrasplenic implantation of KPC/mCherry cells (25,000 cells/spleen) resuspended in 1X PBS following a previously reported protocol [55]. Treatments started 2 days post-implantation, with nanoemulsion doses of 25 mg/kg administered retro-orbitally. Three weeks post-treatment initiation, mice were euthanized and perfused with cold 1X PBS; subsequently, the weights of the tumors and livers were measured and photographed. For the intravenous model, KPC cells were suspended in 1X PBS and then injected into the tail vein of C57BL/6 mice at a concentration of 25,000 cells per injection.

**Peripheral blood mononuclear cells extraction.** Excess huPBMCs were distributed across three 6-well culture plates per donor, utilizing RPMI 1640 complete medium. After 24 h, suspended lymphocytes were removed by washing with 1X PBS and adherent human monocytes were maintained in RPMI 1640 complete medium for following experiments. For human macrophage differentiation, 60 ng/mL of MCSF (PeproTech) was used for M2 polarization as previously described [56].

**Spheroid formation and orthotopic implantation.** For the 3D spheroid model [36], suspensions of PANC1/GFP (1,000 cells/well) and CAFs/mCherry (4,000 cells/well) cells were prepared in DMEM complete medium supplemented with 25 ng/mL of human fibroblast growth factor (FGFb) (Cat no. GF446-100G, Sigma). The resulting mixture was transferred into each well of 96-well ultra-low attachment (ULA) plates. After cell seeding, the plates were centrifuged (200×g, 5 min, RT) and then placed in a humidified atmosphere with 5% CO_2_ at 37°C. On day 3, monocytes from huPBMCs (4,000 cells/well) were added, and spheroid treatment with nanoemulsions (1 mg/mL) was carried out from day 4–7, after which fresh medium was added. On day 10, NOD/ShiLtSz-Prkdc^scid^ (NOD.SCID) mice (Charles River Co) aged 15–20 weeks were orthotopically implanted in the pancreas with 40–50 disaggregated spheroids, which were trypsinized, centrifuged, and resuspended in 50 µL of a 3:2 proportion of Matrigel (Cat no. 354234, Corning):RPMI. Three months after implantation, mice were euthanized to evaluate tumor growth and metastasis using various techniques after animal perfusion with cold 1X PBS.

**Statistical analyses.** Results are presented as means ± standard deviation (SD). To compare more than two sets of data, one-way ANOVA with Dunnett´s multiple comparisons statistical analysis was performed while an unpaired t-student tests was used to analyzed two data sets. All of the statistical analyses were performed with Graph Pad Prism 8. A statistically significant difference was considered as *p < 0.05; **p < 0.01; ***p < 0.001; and ****p < 0.0001, as indicated in the figure legends. For the in vitro experiments, *n* represents the number of experimental repeats. In the in vivo models, *n* refers to the number of animals used in the study, typically originating from at least two separate and independent experiments.

## Results and discussion

**Development and characterization of lipid nanosystems.** In this study, VitE:SM nanosystems were proposed as a strategy to target TAMs in the liver to treat pancreatic cancer metastasis. As mentioned in the introduction, these types of nanocarriers have demonstrated significant potential for delivering therapeutic agents to treat cancer [34], and have proven capable of penetrating dense cancer spheroids, specifically in pancreatic cancer [36, 57]. Likewise, VitE:SM nanoemulsions have shown preferential biodistribution to the liver, and the role of VitE and SM in modulating inflammation has been previously described [46, 47]. The formulated compositions were prepared by adapting an ethanol injection method, enabling a single-step preparation and the straightforward formation of the nanosystems [34, 35, 37] (Fig. 1a). Sphingomyelin (SM) is composed of two long carbon chains (hydrophobic tail), a secondary amine group acting as a linker and a phosphate group (hydrophilic head). Considering its lipophilicity, it is expected that it can form emulsions when combined with oils such as Vitamin E (VitE), a fact that has been validated experimentally and through computational simulation studies [34]. Alternative oils can also form nanoemulsions, in combination with SM, following the same rational and experimental approach. The nanoemulsion compositions could also be complemented by additional phospholipids, including Phosphatidylcholine (PC), Phosphatidylinositol (PI), Phosphatidylserine (PS), Phosphatidylglycerol (PG), or Palmitic acid (PA), since there is growing evidence suggesting that certain phospholipids can influence TAM reprogramming [58, 59]. Consequently, the hydrophobic carbon chain can be incorporated into the oily core of the structure, while the hydrophilic phosphate head is exposed on its surface.

Thus, VitE:SM nanoemulsions and alternative compositions were formulated for comparative purposes, as detailed in Fig. 1b and Table 1. Following formation, all formulations showed appropriate physicochemical properties, with minor differences in size and surface properties, rendering homogeneous populations. In terms of colloidal stability, the formulations were stored at 4°C, and their physicochemical properties, such as particle size, population homogeneity (PDI) and zeta potential were monitored over time. Formulations containing VitE, either with SM or PC (1:0.1 w/w), remained stable for at least 30 days (Figure S1a-b), in agreement with previous results showing high stabilities for VitE:SM nanoemulsions [34].

Subsequently, the lipid nanosystems were incubated with complete RPMI cell culture medium supplemented with 1% FBS at 1:6 (v/v) and measured for up to 24 h at 37°C under orbital shaking. Their size and PDI were determined at different time points by further diluting the sample in ultrapure water at 1:10 (v/v). In line with the results, all formulations showed a good stability with minimal increase in particle size and polydispersity over time (Figure S1c-k).

**The lipid nanosystems comprising VitE:SM demonstrate the most favorable M2 macrophage reprogramming capacity.** Since our primary objective was to reprogram macrophages with nanotechnology, we first assessed the behavior of VitE:SM nanoemulsions in relation to nanosystems with different lipid compositions (Fig. 1b, Table 1) in immortalized murine bone-marrow-derived macrophages (muIBMDM) [60] to determine their potential toxicity and impact on M2 macrophage reprogramming.

**Fig. 2:**
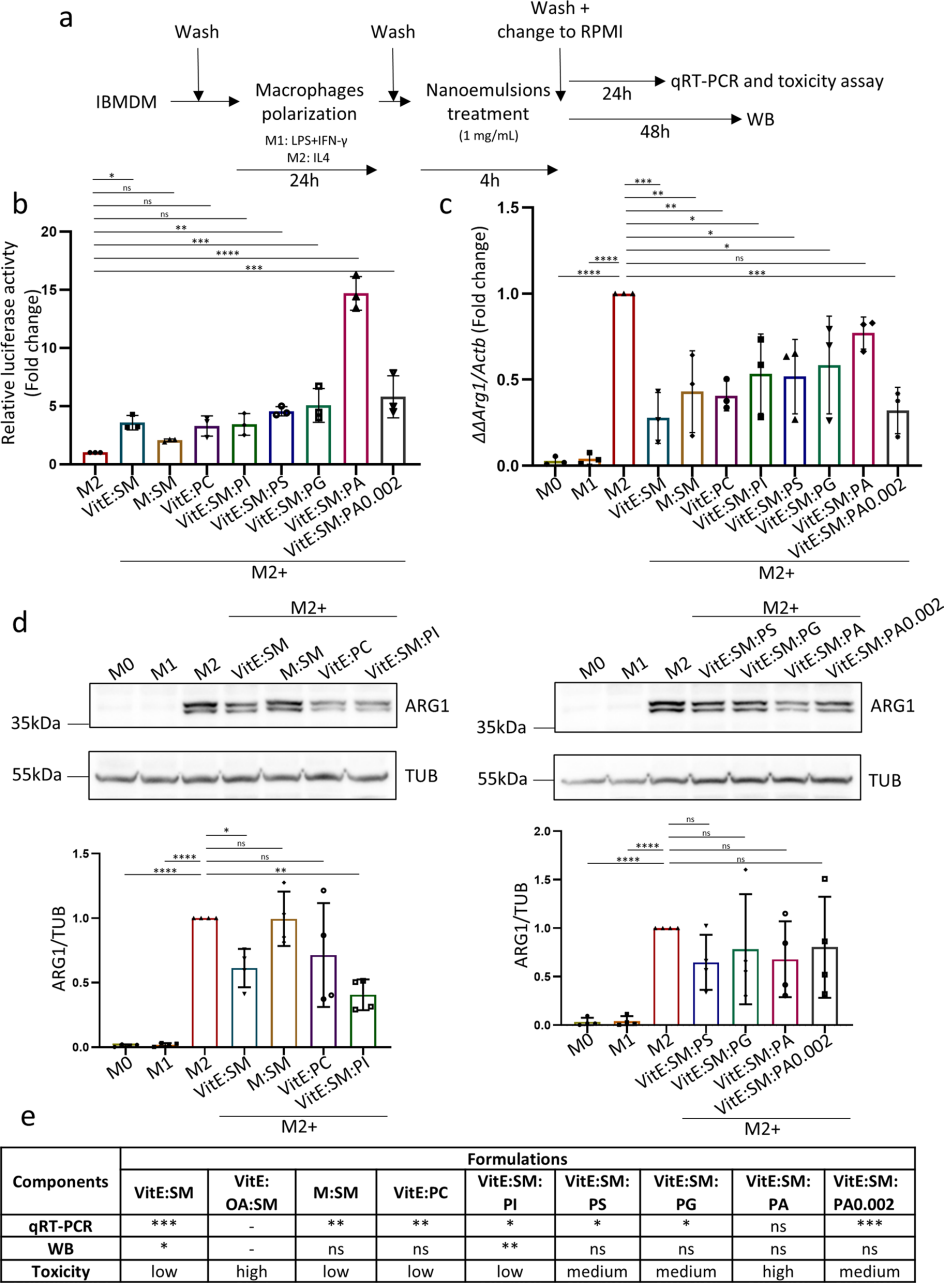
The specific lipid composition of the nanosystems has an influence on M2 macrophage reprogramming. **a** Experimental design for macrophage polarization and treatment with the lipid nanosystems: murine immortalized bone marrow-derived macrophages (IBMDM) were induced to an M1 phenotype using LPS (10 ng/mL) + IFN-γ (10 ng/mL) or to an M2 phenotype with IL4 (10 ng/mL) for 24 h. Following polarization, lipid nanosystems (1 mg/mL) were incubated with macrophages for a 4-h duration: VitE:SM, M:SM, VitE:PC, VitE:SM:PI, VitE:SM:PS, VitE:SM:PG, and VitE:SM:PA at different concentrations of PA. Subsequently, IBMDM cells were washed and cultured in RPMI medium for qRT-PCR and toxicity assays for 24 h, or Western blot analyses for 48 h. **b** Fold change in relative luciferase activity (i.e., toxicity) ± SD determined in IBMDM cultures treated with the indicated different nanoemulsion compositions compared to untreated M2 polarized macrophages, set as 1.0 (n = 3). **c** Analysis of the levels of the principal M2 marker *Arg1* by qRT-PCR. Bars represent the mean fold change ± SD (n = 3), with untreated M2 set as 1.0. **d** Top: Representative Western immunoblots of ARG1 protein expression levels. Bottom: Densitometric analysis of the immunoblots is represented in the bar diagram. Bars represent the mean fold change ± SD (n = 4), with untreated M2 set as 1.0. **e** Summary table of three parameters chosen for evaluating the best nanoemulsion composition. For toxicity, low < 4 AU, medium = 4–7 AU, high > 7 AU. ∗  = p < 0.05; ∗  ∗  = p < 0.01; ∗  ∗  ∗  = p < 0.001; ∗  ∗  ∗  ∗  = p < 0.0001; ns = not significant. One-way ANOVA test for multiple comparisons with Dunnett’s post hoc test, compared to the untreated M2 sample

IBMDM macrophages were polarized for 24 h with LPS + IFN-γ for M1 polarization and IL-4 to induce M2 polarization. IL-4 is the most common cytokine used to polarize macrophages to an M2 state [61–63]; however, it is important to note that M2 polarization of macrophages is co-regulated by a variety of cytokines and chemokines released by PDAC cells and CAFs in the TME [15]. Following polarization, M2 macrophages were treated with nanoemulsions for 4 h to assess toxicity and reprogramming by qRT-PCR analysis after a 24-h post-treatment period, and by Western blotting to evaluate the expression of the principal M2 marker Arg1 at 48 h post-treatment (Fig. 2a). To assess associated toxicity induced by the different formulations, a bioluminescent assay that measures the release of adenylate kinase from damaged cells was employed, revealing that VitE:SM was one of the least cytotoxic formulations (Fig. 2b), which was confirmed by light microscopy (Figure S2a). VitE:OA:SM was discarded due the high cytotoxicity observed (Figure S2a).

Regarding reprogramming towards a more M0/M1 phenotype, qRT-PCR and Western Blot analysis of Arginase 1 (*Arg1*/ARG1) expression, a well-known marker for M2 macrophages in PDAC [64] was performed (Fig. 2a). By qRT-PCR analysis we observed a general decrease in *Arg1* expression with all the tested formulations, with a more pronounced decrease observed in the case of the VitE:SM formulation compared to untreated M2 macrophages (Fig. 2c). This trend was similarly observed at the protein level. While a decrease in ARG1 expression was achieved with nearly all the formulations, it was only statistically significant in the case of nanoemulsions containing VitE:SM and VitE:SM:PI (Fig. 2d).

Based on these data, we determined that the most effective formulation for macrophage reprogramming and the most suitable low toxic composition for further development was the nanosystem containing VitE:SM (Fig. 2e). To investigate the potential synergies offered by VitE and SM in TAM reprogramming, we conducted an additional experiment in which IBMDM macrophages were first polarized with IL-4 for 24 h to induce M2 polarization. The M2 macrophages were then treated for 4 h with the VitE:SM nanoemulsions, SM, VitE, or a free combination of VitE and SM to assess toxicity (% live cells) and M2 reprogramming via flow cytometry (CD45 + <  CD11b +  < F4/80 +  < **CD206 +**) 24 h post-treatment (Figure S2b). Our results confirm that the VitE:SM nanoparticle composition exhibits a synergistic effect to reprogram M2 TAMs and demonstrates improved cell viability compared to the other compositions. To further analyze the structure of this composition, we employed field emission scanning electron microscopy (Figure S3).

We then investigated whether primary murine macrophages derived from primary bone marrow monocytes (BMDM) treated with VitE:SM maintained their reprogramming over time and whether these effects were reversible upon subsequent exposure to the M2-polarizing cytokine IL-4 (Figure S4a). Indeed, Western blot analysis of ARG1 revealed a continued reduction of this M2 marker over time in BMDMs treated with the VitE:SM nanosystem. Additionally, VitE:SM nanoemulsion-treated macrophages that were repolarized with IL-4 did not increase ARG1 to levels observed in control groups (Figure S4b). Considering the implications for in vivo use, these findings suggest that despite the continuous influence from tumor secreted pro-TAM factors, treated macrophages are likely to retain the polarization state induced by the VitE:SM nanoemulsions over time; however, as stated above, M2 polarization of macrophages in vivo is co-regulated by a variety of cytokines and chemokines released by PDAC cells and CAFs in the TME [15].

We hypothesized that the potent reprogramming capacity of the VitE:SM formulation arises from two key properties. On the one hand, it is well-established that the phagocytosis of lipid membranes by macrophages affects their programming and metabolism [32, 65, 66]. Some studies utilize cell membrane-derived nanoparticles (nanoghosts) for macrophage reprogramming, attributing this effect to components like cytokines and chemokines present in the cell membranes of nanoghosts. However, it is also plausible that membrane sphingolipids such as SM play a crucial role in macrophage reprogramming [44–47, 66]. On the other hand, the immunostimulant properties of VitE may also contribute to macrophage reprogramming [40–42]. Thus, the dual actions of VitE and SM suggests a potential synergy in enhancing the reprogramming capacity of this composition, making this combination particularly promising for further experimentation.

**Fig. 3:**
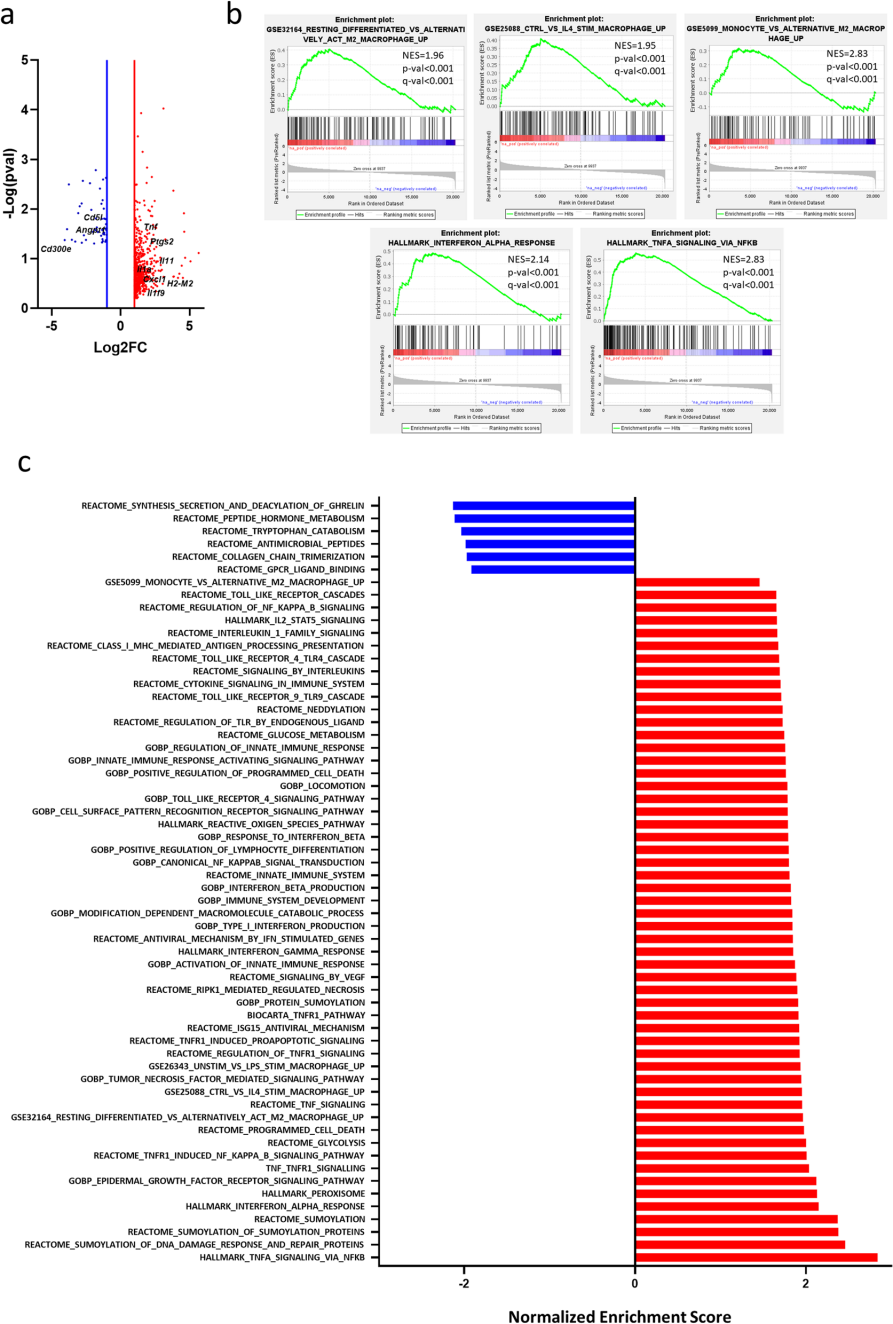
RNAseq analysis of M2 and M2 VitE:SM nanoemulsion-treated macrophages. **a** Volcano plot showing differentially expressed genes in VitE:SM nanoemulsions-treated (1 mg/mL) M2 macrophages versus naïve M2 macrophages. Red dots, genes upregulated; blue dots, genes downregulated. Genes of interested have been labelled in black. **b** Gene set enrichment analysis (GSEA) plots showing a significant enrichment in the indicated gene signatures for VitE:SM nanoemulsions-treated M2 macrophages. Normalized enrichment score (NES) and p- and q- values are indicated for each plot. **c** Histogram representation of the NES of significantly altered gene signatures down- (blue) and upregulated (red) in VitE:SM-treated M2 macrophages

**RNAseq analysis of M2 and M2 VitE:SM nanoemulsion-treated macrophages.** To further investigate the effects of the VitE:SM formulation on M2 macrophage polarization at the transcriptomic level, we performed RNAseq on IL-4-stimulated (M2) and IL-4-stimulated VitE:SM nanoemulsion-treated muBMDMs. Among the genes significantly upregulated in VitE:SM-treated macrophages, we found genes related to inflammation (i.e.*, Tnf, Ptgs2, Il-11, Il-1a, Cxcl1*) and with antigen presentation (i.e., *H2-*M2). Additionally, among the downregulated genes, we identified *Cd51* and *Cd300e,* which are related to M2 polarization, and *Angpt1* which is a pro-angiogenic factor (Fig. 3a).

We next performed a Gene Set Enrichment Analysis (GSEA) employing Molecular Signature Database (MsigDB) signatures (including Gene Ontology, Reactome and Hallmarks) or custom gene signatures. Interestingly, VitE:SM nanoemulsion-treated macrophages recover a pattern related to resting differentiated macrophages or monocytes, rather than a gene set associated with M2 or IL-4 stimulated macrophages. We also found mainly an enrichment in gene signatures related to TNF-TNFR1 signaling, but also macrophage activation via Toll receptors, interferon-related signaling, reactive oxygen species and signatures related to innate immune system activation, indicating that VitE:SM treatment induces a reprogramming of M2 macrophages towards a more inflammatory and less immunosuppressive phenotype, which is in line with our previous results and hypothesis (Fig. 3b-c).

**Fig. 4:**
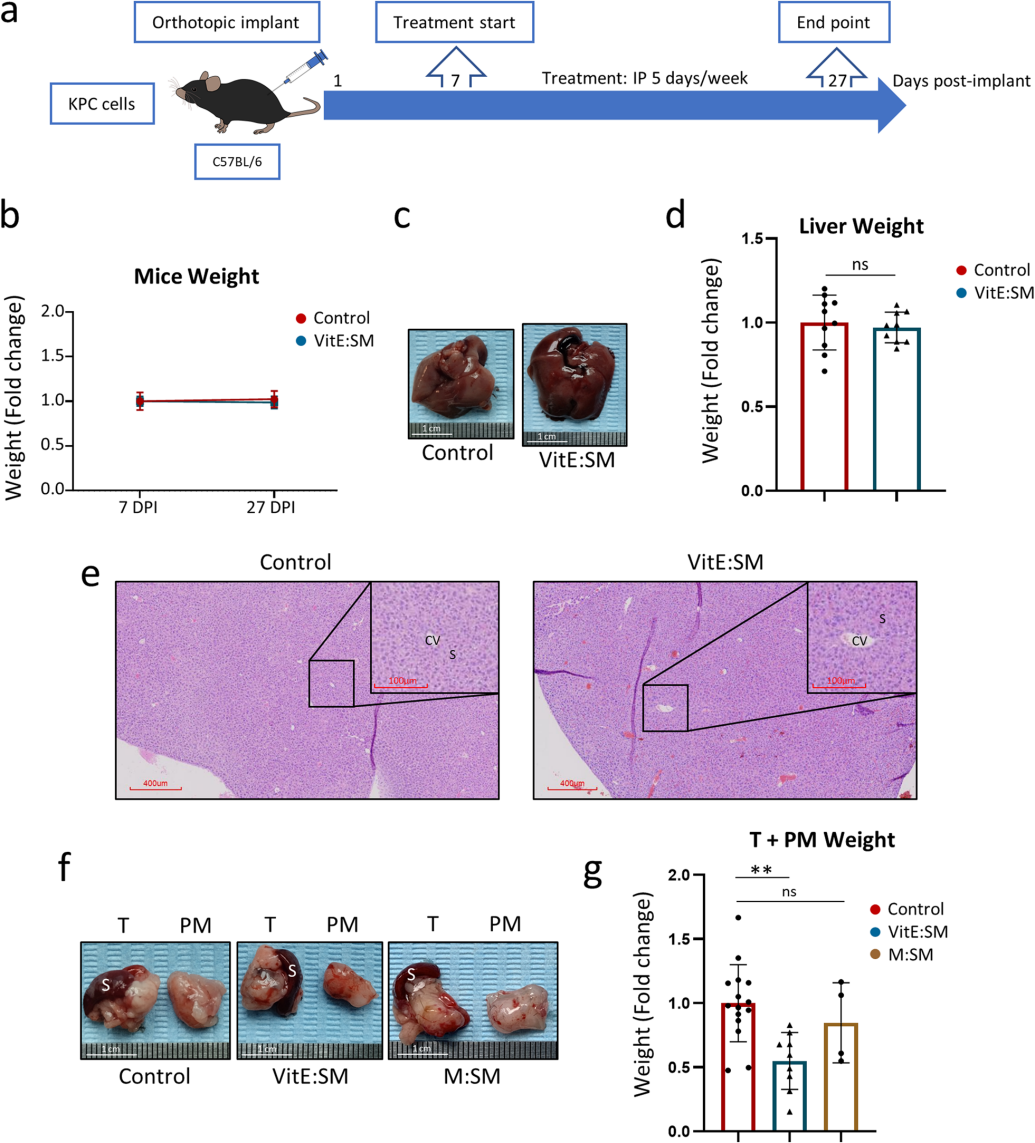
Intraperitoneal injection of VitE:SM nanoemulsions reduces tumor burden in an orthotopic KPC tumor model. **a** Experimental set-up for the in vivo syngeneic model in C57BL/6 mice. Mice were orthotopically implanted with 2,500 KPC cells/pancreas and treatment was initiated 7 days post-implantation, receiving VitE:SM nanoemulsions treatments (69 mg/kg) 5 days per week for three weeks via intraperitoneal (IP) injection. **b** Fold change ± SD of the total weight of the animals at the start and completion of the treatment. The initial weights for the control (n = 10) and VitE:SM (n = 9) groups from two independent experiments were pooled and each set as 1.0 to observe the weight evolution throughout the experiment. Days post-implantion (DPI). **c** Representative images of the liver at the experimental endpoint from control (n = 10) and VitE:SM-treated (n = 9) groups from two independent experiments. Scale = 1cm. **d** Liver weight from **c**. Data were normalized for each independent experiment (n = 2) with the bars representing the mean fold change ± SD, with the control set as 1.0. Unpaired t test. ns = not significant. **e** Representative images (40X) of H&E-stained liver slices at the experimental euthanize time point from control or VitE:SM-treated mice. Scale = 400µm. Zoom areas are depicted within squares. Scale = 100µm. *CV* Central vein, *S* Sinusoids. **f** Representative images of the tumor (T) and adjacent peritoneum tumor-derived masses (PM) at the experimental endpoint for control (n = 10) and VitE:SM-treated (n = 9) groups from two independent experiments. An additional control group treated with non-effective nanoparticles M:SM (n = 4) was included. All tumor images are presented in Figure S6. Scale = 1cm. S = Spleen served as an anatomical reference for the pancreas. **g** Fold change in T + adjacent PM weight ± SD from **f**. Data were normalized for each duplicate experiment with the control set as 1.0. One-way ANOVA with Dunnett’s post hoc test, compared to control. ∗  ∗  = p < 0.01. ns = not significant

**The intraperitoneal injection of VitE:SM nanoemulsions reduces tumor burden in an orthotopic KPC tumor model.** To initially validate the in vivo efficacy of the nanosystems, we conducted a preliminary study employing murine PDAC cells obtained from spontaneous tumors from the KPC (LSL-Kras^G12D/+^; LSL-Trp53^R172H/+^; Pdx-1-Cre) mouse model [67]. These cells were orthotopically injected into the pancreas of syngeneic and immunocompetent C57BL/6 wild-type (WT) mice (Fig. 4a), with the nanosystem treatment commencing on the seventh day post-implantation in two distinct non-parallel experiments. In each experiment, randomized groups of 4 or 5 mice were intraperitoneally injected with a vehicle solution (0.9% NaCl) for the control mice or 69 mg/kg of VitE:SM nanoemulsions for treated mice. This regimen was applied for five consecutive days per week. Mice were euthanized on day 27 post-implantation, after confirming tumor growth in a sentinel mouse by Magnetic Resonance Imaging (MRI) (Figure S5a). Total body weight was recorded at the start and end of the treatment to assess nanoemulsion toxicity, revealing no differences between groups throughout the study period (Fig. 4b). In this model, tumor presence typically impairs weight gain and often leads to reduced food consumption or tumor-associated cachexia, so the lack of weight gain is expected. Full-body necropsy was then performed and no liver toxicity was observed at the organotypic level (Fig. 4c), nor were differences observed in liver weight (Fig. 4d). At the histopathological level, examination of H&E-stained liver slides showed that the structure and architecture of the liver remained unaffected by the treatment. There was no evidence of inflammation, necrosis, or loss of architecture in the central vein or sinusoids. (Fig. 4e). Finally, the assessment of liver toxicity following treatment with VitE:SM nanoemulsions, as determined by liver function tests, did not reveal elevated values of ALT, AST and GGT when compared to standard reference ranges (Table S3). Liver toxicity was also absent in non-implanted mice treated with three doses of VitE:SM nanoparticles administered every two days over a one-week period. Serum analysis after multiples treatments showed normal ALT enzyme levels, with no significant difference between control and VitE:SM-treated mice (Figure S5b). Additionally, the mice did not experience weight loss during the treatment process (Figure S5c). This underscores the biocompatibility of the chosen nanosystem, as evidenced by the absence of toxicity associated with the nanoemulsion. Moreover, unlike other treatments that deplete TAMs, such as clodronate or toxin-conjugated monoclonal antibodies [31, 68], VitE:SM nanosystems could offer a safer therapeutic option.

Both at the level of the tumor alone (Figure S5d) or including the adjacent peritoneal tumor-derived masses (Fig. 4f-g), a reduction in total tumor weight was evident in mice treated with the VitE:SM nanosystem compared to the control group (Fig. 4f-g and S6). Importantly, and to specifically highlight the activity of the VitE:SM formulation, an additional nanoemulsion control group was included. Specifically, mice were also treated with Mygliol (M):SM nanoemulsion, which were shown to be less effective at the level of M2 TAM reprogramming (Fig. 2). Tumor weights confirmed a significant decrease in tumor burden with intraperitoneal administration of VitE:SM nanoemulsions but not with the M:SM nanoparticles (Figure S5d and 4g), indicating that the VitE:SM formulation has specific anti-tumoral properties.

Taken together, these findings align with other studies that have also utilized nanoparticles to modulate the immune system; however, the composition of the nanoemulsion appears to be critical to achieve an anti-tumor effect. Thus, the integration of nanotechnology to enhance immune responses and/or affect tumor growth represents a growing and consistent trend and highlights the efficacy of nanoparticle-based approaches in immuno- and tumor modulation [69, 70].

**Fig. 5:**
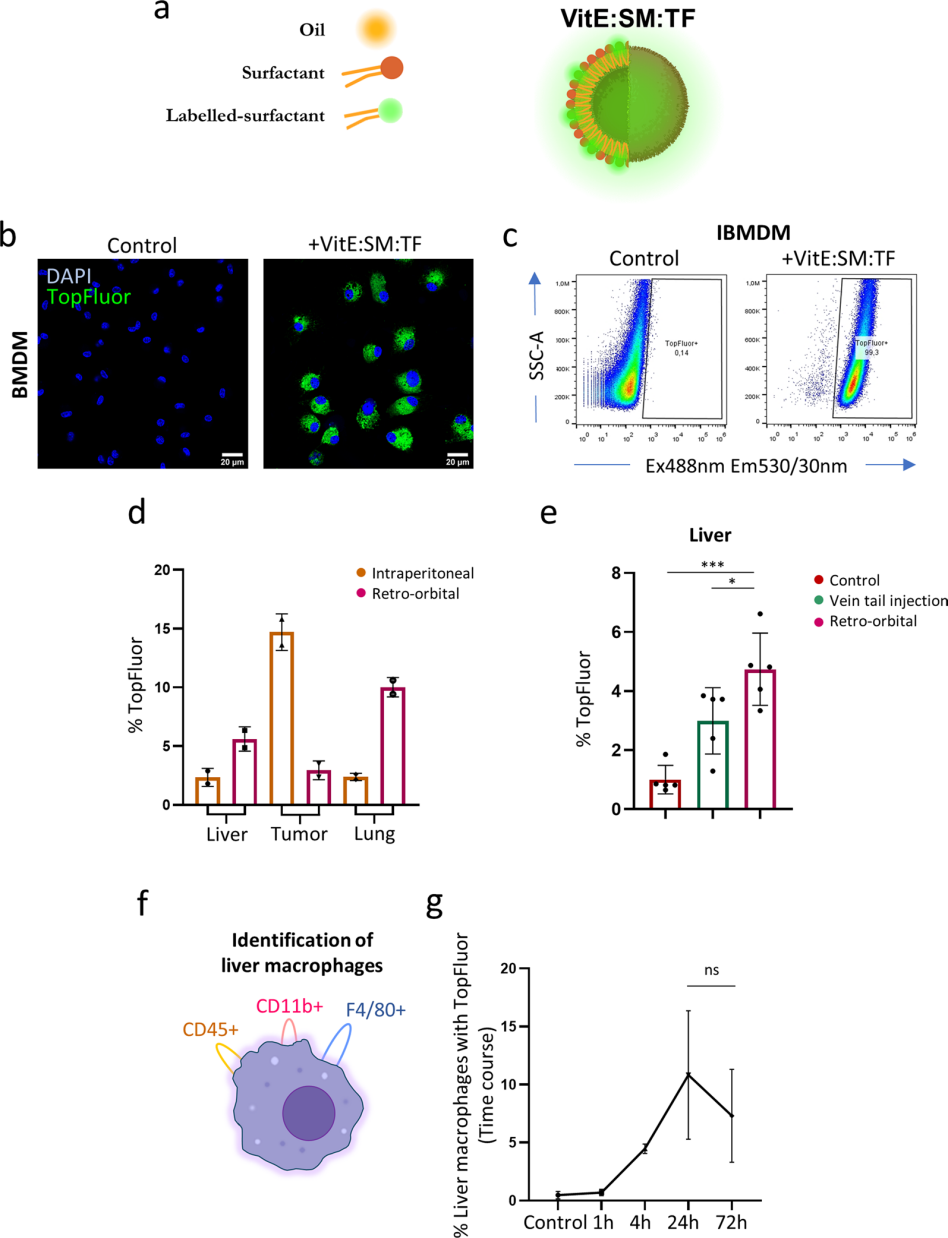
Targeting capacity of the TopFluor® (TF) labelled VitE:SM nanoemulsions in vitro and in vivo. **a** TopFluor® was incorporated in the surfactant (TopFluor®-labelled-sphingomyelin) and injected in the aqueous phase as previously explained (Fig. 1a). VitE:SM:TF = Vitamin E + TopFluor®-labelled-sphingomyelin nanosystem. **b** Confocal images of muBMDM cells treated or not with TF-labelled nanoemulsions (VitE:SM:TF, 0.5 mg/mL). Scale bar = 20 µM. DAPI: blue, TF: green. **c** Cytometry dot plots of muIBMDM cells in the presence or absence of VitE:SM:TF (0.5 mg/mL) nanoemulsions. Murine IBMDMs containing VitE:SM:TF nanoemulsions were detected at a wavelength of Ex488 nm Em530/30 nm. SSC-A: Side Scatter (Area). **d** Flow cytometry analysis of the percentage of TopFluor®-positive live cells ± SD in three different organs (liver, tumor, and lung) under two treatment modalities: intraperitoneal or retro-orbital (n = 2 mice per treatment). **e** Flow cytometry analysis of the percentage of TopFluor®-positive live cells ± SD in the liver under two intravenous treatment modalities (tail vein injection or retro-orbital) compared to control (n = 5 control, n = 5 mice per each treatment). ∗  = p < 0.05; ∗  ∗  ∗  = p < 0.001. One-way ANOVA with Dunnett’s post hoc test, compared to the retro-orbital condition. **f** Schematic representation of the markers used to identify macrophages: CD45 + , CD11b + and F4/80 + . **g** Flow cytometry analysis of the percentage of macrophages (CD45 + , CD11b + and F480 +) ± SD targeted by the VitE:SM:TF nanoemulsions over time, with data collected at 1 h, 4 h, 24 h and 72 h. Unpaired t test comparing 24 and 72 h. *ns*  not significant

**Targeting capacity of the TopFluor® (TF)-labelled VitE:SM nanoemulsions in vitro and in vivo.** To further evaluate the in vivo application of the nanosystems, nanoemulsions were formulated using SM labeled with the green fluorophore TopFluor® (TF) for tracking purposes in mice (Fig. 5a and Table 2). First, confocal microscopy images of IBMDM macrophages revealed green fluorescence in those cultures treated with VitE:SM:TF nanoemulsions (Fig. 5b). Detection of the fluorophore by flow cytometry, using Ex488nm Em530/30nm, showed nearly complete staining of macrophages (Fig. 5c). Since the goal of this study was to target liver TAMs, we assessed which route of administration would favor liver biodistribution, as measured by flow cytometric analysis of tumors, liver and lungs. VitE:SM:TF nanoemulsions were administered intraperitoneally or retro-orbitally to KPC orthotopically implanted C57BL/6 mice seven days post-surgery, at a dose of 25 mg/kg of VitE:SM:TF nanoemulsions. Mice were euthanized 48 h later to assess TF biodistribution in the tumor and the two principal organs susceptible to develop metastasis: liver and lungs. Following organ digestion, we confirmed by flow cytometry that while intraperitoneal administration is optimal for reaching the tumor, retro-orbital injection is more effective in reaching the liver and the lungs (Fig. 5d). To further compare retro-orbital injection with another intravenous route, such as tail vein injection, we conducted an additional biodistribution experiment, which confirmed that retro-orbital injection was more efficient in delivering the treatment to the liver, the primary target organ of this study (Fig. 5e). To determine the kinetics of VitE:SM:TF nanoemulsion liver targeting, and specifically liver macrophage (i.e., CD45 + , CD11b + , and F4/80 +) targeting (Fig. 5f), VitE:SM:TF fluorescence was assessed 1 h, 4 h, 24 h, and 72 h post retro-orbital injection. Flow cytometry analysis of digested livers indicated that peak accumulation of VitE:SM:TF in liver macrophages was reached at 24 h, with a decrease at 72 h (Fig. 5g). Thus, we established that the optimal treatment regimen for subsequent in vivo intervention studies to be retro-orbital injection every 48 h.

**Table 2:** Physicochemical properties of VitE:SM, VitE:SM:TF and VitE:SM:LY nanosystems measured, after preparation, by dynamic light scattering (DLS) and laser doppler anemometry (LDA) including hydrodynamic size, polydispersity index (PDI), zeta potential (ZP) and encapsulation efficiency (%EE) of LY. Results are expressed as mean ± SD, n = 5

Formulation	Size (nm)	PDI	ZP (mV)	%EE LY
VitE:SM	97 ± 5	0.08 ± 0.02	− 13 ± 8	–
VitE:SM:TF	102 ± 4	0.07 ± 0.03	− 6 ± 1	–
VitE:SM:LY	107 ± 5	0.09 ± 0.02	− 12 ± 2	92 ± 2

*nm* nanometer, *PDI* polydispersity index,* ZP* zeta potential in millivolts (mV)

**Fig. 6:**
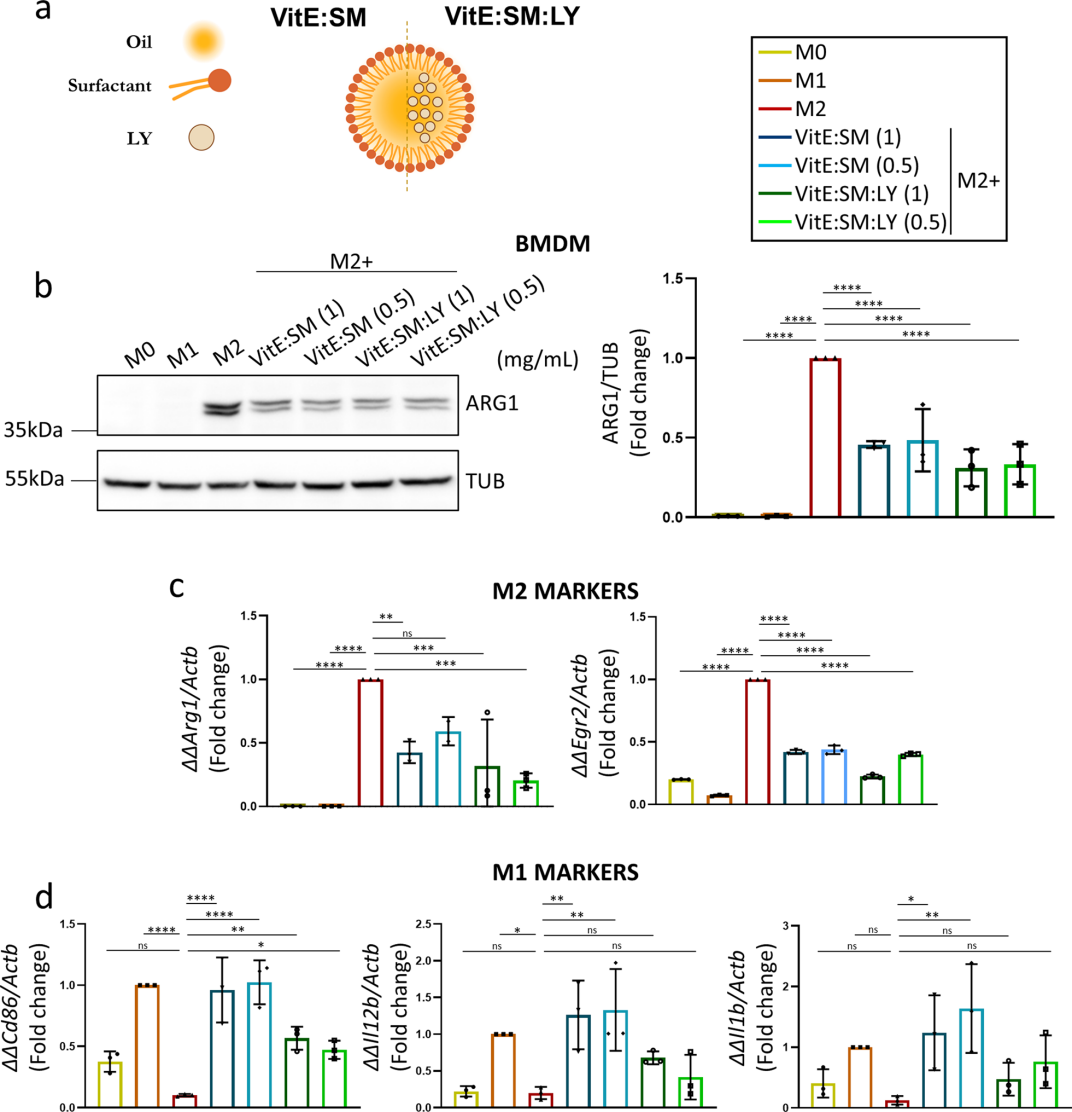
Testing VitE:SM nanoemulsions loaded with the TGF-βR1 inhibitor (LY2157299). **a** For LY loading, the TGF-βR1 inhibitor was dissolved in the organic phase and injected in the aqueous phase as previously explained (Fig. 1a). VitE:SM = Vitamin E + sphingomyelin nanosystem; VitE:SM:LY = Vitamin E + sphingomyelin + encapsulated TGF-βR1 inhibitor. **b** Left: Western immunoblot analysis of ARG1 protein levels in muBMDMs polarized as in Fig. 2a to M0, M1 or M2 and treated with empty or LY encapsulated VitE:SM nanoemulsions at two different concentrations: 1 and 0.5 mg/mL. Right: Densitometric analysis of the immunoblots, with the bars representing the mean fold change ± SD (n = 3), with untreated M2 set as 1.0. **c** Analysis by qRT-PCR of two M2 markers, *Arg1* and *Egr2*, in the same samples as described in **b**. Bars represent the mean fold change ± SD (n = 3), with untreated M2 set as 1.0. **d** Analysis by qRT-PCR of M1 markers, *Cd86*, *Il12b* and *Il1b*, in the same samples as in **b** and **c**. Bars represent the mean fold-change ± SD (n = 3), with untreated M1 set as 1.0. ∗  = p < 0.05; ∗  ∗  = p < 0.01; ∗  ∗  ∗  = p < 0.001; ∗  ∗  ∗  ∗  = p < 0.0001; *ns*  not significant. One-way ANOVA with Dunnett’s post hoc test, compared to M2 in **b** and **c** and to M1 in **d**

**Loading of VitE:SM nanoemulsions with the TGF-βR1 inhibitor (LY2157299) reduces tumor growth and diminishes TAM liver infiltration.** Next, we decided to further explore the potential of VitE:SM nanoemulsions to encapsulate and deliver the TGF-βR1 inhibitor Galunisertib LY2157299, referred to as LY (VitE:SM:LY) (Fig. 6a and Table 2). We hypothesized that LY would inhibit the activity of fibrotic cytokines in vivo and prevent the M2 polarization of macrophages via TGF-β [71–73], as previously discussed, thereby further obstructing the formation of the pre-metastatic niche in the liver [48–50]. Moreover, the encapsulation of LY into the VitE:SM nanosystem should in theory enhance its delivery and mitigate non-specific toxicity often associated with the administration of free LY [74, 75]. We first tested the VitE:SM and VitE:SM:LY compositions at different concentrations (1 and 0.5 mg/mL) in muBMDM cells polarized to an M2 state with IL-4. Western blot analysis of ARG1 protein levels revealed a decrease in this M2 marker across all treated samples, with no significant differences observed between different concentrations or with LY encapsulation (Fig. 6b). Additionally, qRT-PCR analysis of various M1 or M2 markers consistently demonstrated a decrease in classical M2 markers such as *Arg1* and *Egr2* (Fig. 6c) and an increase in some M1 markers such as *Cd86*, *Il1b*, and *Il12b* (Fig. 6d). Of note, the addition of LY to the nanoemulsions did not lead to an improvement in macrophage reprogramming, which is in slight contrast to what we have previously seen with another TGF-βR1 inhibitor in human M2-polarized macrophages [56]. We speculated that the potent effect of the VitE:SM vehicle was masking the influence of LY in vitro, but we also reasoned that the additive effect offered by encapsulating LY in the nanosystems would be more evident in vivo.

**Fig. 7:**
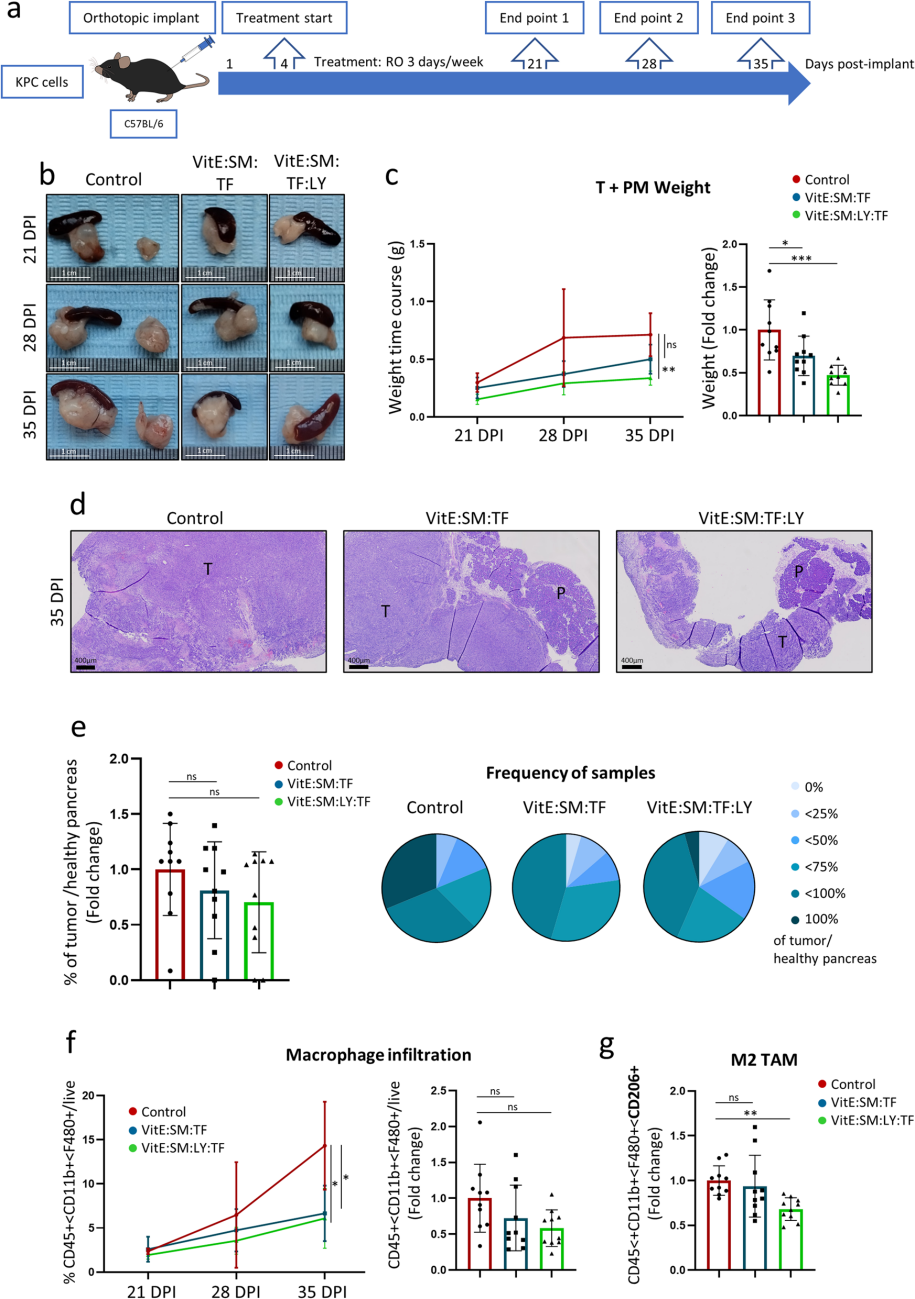
TGF-βR1 inhibitor (LY2157299)-loaded VitE:SM nanoemulsions reduce tumor growth and diminish TAM liver infiltration. **a** Schematic representation of the retro-orbital in vivo treatment experiment. C57BL/6 mice were orthotopically implanted with 2,500 KPC cells/pancreas and retro-orbital treatment (25 mg/kg) was initiated on day 4 post-implantation and administered 3 days per week until the euthanize end points: 21, 28, or 35 days post-implantion. **b** Representative macroscopic images of tumors and adjacent PM for the three groups (Control, VitE:SM:TF and VitE:SM:LY:TF) at the euthanize end points: 21, 28, or 35 days post-implantion (DPI). Scale = 1cm. **c** (Left panel) Weight (g) time course of tumors and adjacent PM from b) at the three experimental end points. Three animals per group (n = 3) were euthanized at 21 and 28 DPI and four (n = 4) at 35 DPI. (Right panel) Fold change in tumor and adjacent PM weight (g) for all animals across the entire experimental time course. Bars represent the mean fold change ± SD (n = 10), with control set as 1.0. One-way ANOVA test for multiple comparisons with Dunnett’s post hoc test, compared to Control. **d** Representative images (40X) of H&E-stained tumors from control, VitE:SM:TF- or VitE:SM:LY:TF-treated mice on 35 DPI. T = Tumor, P = Pancreas. Scale = 400µm. **e** (Left panel) Ratio of the % area of tumor/healthy pancreas compared to the total histologic pancreas/tumor area quantified in the H&E-stained sections from **d**. One-way ANOVA test for multiple comparisons with Dunnett’s post hoc test, compared to Control set as 1.0. (Right panel) Frequency of samples from d) classified based on different percentages of tumor/healthy pancreas as defined in the legend. **f** Flow cytometry analysis of the percentage of CD45 +  < CD11b +  < F480 + macrophages in the liver samples from c. (Left panel) Time course of macrophage infiltration in the liver at the three euthanize end points: 21, 28 or 35 DPI. One-way ANOVA test for multiple comparisons with Dunnett’s post hoc test, compared to Control set as 1.0. (Right panel) Fold change in liver macrophage infiltration for all animals in the experiment. Bars represent the mean fold-change ± SD (n = 10), with Control set as 1.0. One-way ANOVA test for multiple comparisons with Dunnett’s post hoc test, compared to Control. **g** Flow cytometry analysis of the M2 TAM marker CD206. Bars represent the mean fold-change ± SD (n = 10), with Control set as 1.0. One-way ANOVA test for multiple comparisons with Dunnett’s post hoc test, compared to Control. ∗  = p < 0.05; ∗  ∗  = p < 0.01; ∗  ∗  ∗  = p < 0.001; ∗  ∗  ∗  ∗  = p < 0.0001; ns = not significant

Towards this end, C57BL/6 mice were orthotopically implanted with KPC cells, and VitE:SM:TF or VitE:SM:LY:TF nanoemulsion treatment was initiated on day 4 post-implantation at a dose of 25 mg/kg, administered retro-orbitally every 48 h (3 days per week). Three euthanize time points to assess tumor progression were established: days 21, 28, and 35 post-implantation (Fig. 7a). At the indicated time points, tumors were extracted, and a reduction in tumor size in mice treated with empty nanoemulsions (VitE:SM:TF) and with nanoemulsions loaded with LY (VitE:SM:LY:TF) was observed (Fig. 7b). Monitoring tumor weight over time revealed that VitE:SM:TF and, more significantly, VitE:SM:LY:TF exerted a cytostatic effect compared to control mice (Fig. 7c). Likewise, the sum of the data across all time points collectively exhibited a significant reduction in tumor weight in mice treated with both nanoemulsion formulations (Fig. 7c). Histological analysis of tumor samples revealed a higher proportion of tumoral area in mice from the control group, whereas areas of healthy pancreas were still prominent in many mouse pancreata from the treatment groups (Fig. 7d-e).

Despite attempts to detect liver metastases in the orthotopic model, no macroscopic metastases were found at the experimental endpoint (d35) (Figure S7a) in any group, nor did our histological analyses of the liver reveal evident areas of tumor micrometastases (Figure S7b). Since our goal was to target liver macrophages in the hope of reprogramming TAMs, livers pieces were digested for flow cytometric analysis, revealing that not only did the nanosystems reach the macrophages in the liver (Figure S7c), but a reduction in the total population of CD45 + /CD11b + /F4/80 + infiltrated macrophages with nanoemulsion treatments was observed (Fig. 7f). Moreover, we analyzed the macrophage population characterized by CD206 expression, a parallel M2 marker for flow cytometry, and found a significant reduction in these TAMs with LY encapsulated nanoemulsions (Fig. 7g), reinforcing our objective of M2 TAM targeting and reprogramming in the liver. While our data support macrophage reprogramming as the mechanism of action by which the VitE:SM nanoemulsions affect tumor growth and TAM liver infiltration, we cannot rule out a possible direct effect on tumor cells; although, flow cytometry analysis showed a preferential accumulation in macrophages versus the rest of live cells (Figure S7d).

To explore the immune profiling and metastasis-related cytokine response induced by nanoparticle treatment, we extracted RNA from liver and tumor tissues and performed qRT-PCR analysis on key cytokines. In both the liver (Figure S7e) and tumor (Figure S7f), treatment with VitE:SM, and especially VitE:SM:LY, led to a significant upregulation of pro-inflammatory and Th1-type cytokines (*Il12, Tnf, Ptgs2, Cxcl1, Gzmb*, and *Il6*), which are crucial for activating immune cells like macrophages and T cells. This pro-inflammatory shift was further supported by the downregulation of immunosuppressive cytokines (*Arg1, Pglyrp1, Ccl2*, and *Mpo*), contributing to a less permissive environment for metastasis. These findings align with previous studies using Galunisertib LY2157299, which also showed a shift towards a pro-inflammatory, anti-tumor immune response [76]. Additionally, these in vivo results are consistent with in vitro data detailed above (Figs. 2 and 3), where VitE:SM and VitE:SM:LY effectively reprogrammed immunosuppressive M2 macrophages into a pro-inflammatory M0/M1 phenotype.

In contrast to the above detailed in vitro studies (Fig. 6), these in vivo results highlight the synergistic impact of incorporating LY into the VitE:SM nanoemulsions. The results reveal superior outcomes with LY encapsulation compared to the effectiveness of VitE:SM nanosystems alone across all evaluated parameters, including tumor growth, the percentage of healthy pancreas in histological samples, and the reprogramming and infiltration of TAMs in the liver. This enhanced efficacy may be attributed to the physiologically relevant environment obtained in the in vivo setting where TGF-β signaling plays a crucial role in tumor progression and TAM recruitment to the liver [16, 17]. This underscored the significance of utilizing the LY-loaded VitE:SM nanoemulsions for our subsequent in vivo PDAC metastasis studies.

**TGF-βR1 inhibitor (LY2157299)-loaded VitE:SM nanoemulsions diminish liver metastasis in an intrasplenic KPC metastasis model.** While the orthotopic model was an excellent and biologically-relevant model to study the effect of the nanoemulsions on primary tumor growth, we concluded that it was not the most suitable option for assessing liver metastasis, as the progression of the primary tumor was too rapid and precluded the development of macroscopic liver metastases within the time frame of the study (i.e., before humane endpoint of euthanasia). Consequently, we considered the feasibility of testing our treatment in a more appropriate metastasis model. In general, there are three models for studying PDAC metastasis: intravenous, intraperitoneal, and intrasplenic models, commonly utilized for investigating lung, peritoneal and lymph node, and liver metastases, respectively [77]. As our objective was to reprogram TAMs in the liver to prevent metastasis, the intrasplenic model [55, 78] represented a better option for our nanosystems, as injected cells have direct communication with the liver through the splenic vein (SV). However, a pilot experiment was conducted to compare intrasplenic versus intravenous injection of KPC cells in order to ascertain the most suitable model for validating our nanosystems. Consistent with findings in the literature [77], PCR analysis revealed a higher number of CRE DNA copies (derived from KPC cells) in the liver when using the intrasplenic model. Conversely, the intravenous model emerged as a more favorable option for promoting lung colonization (Figure S8).

**Fig. 8:**
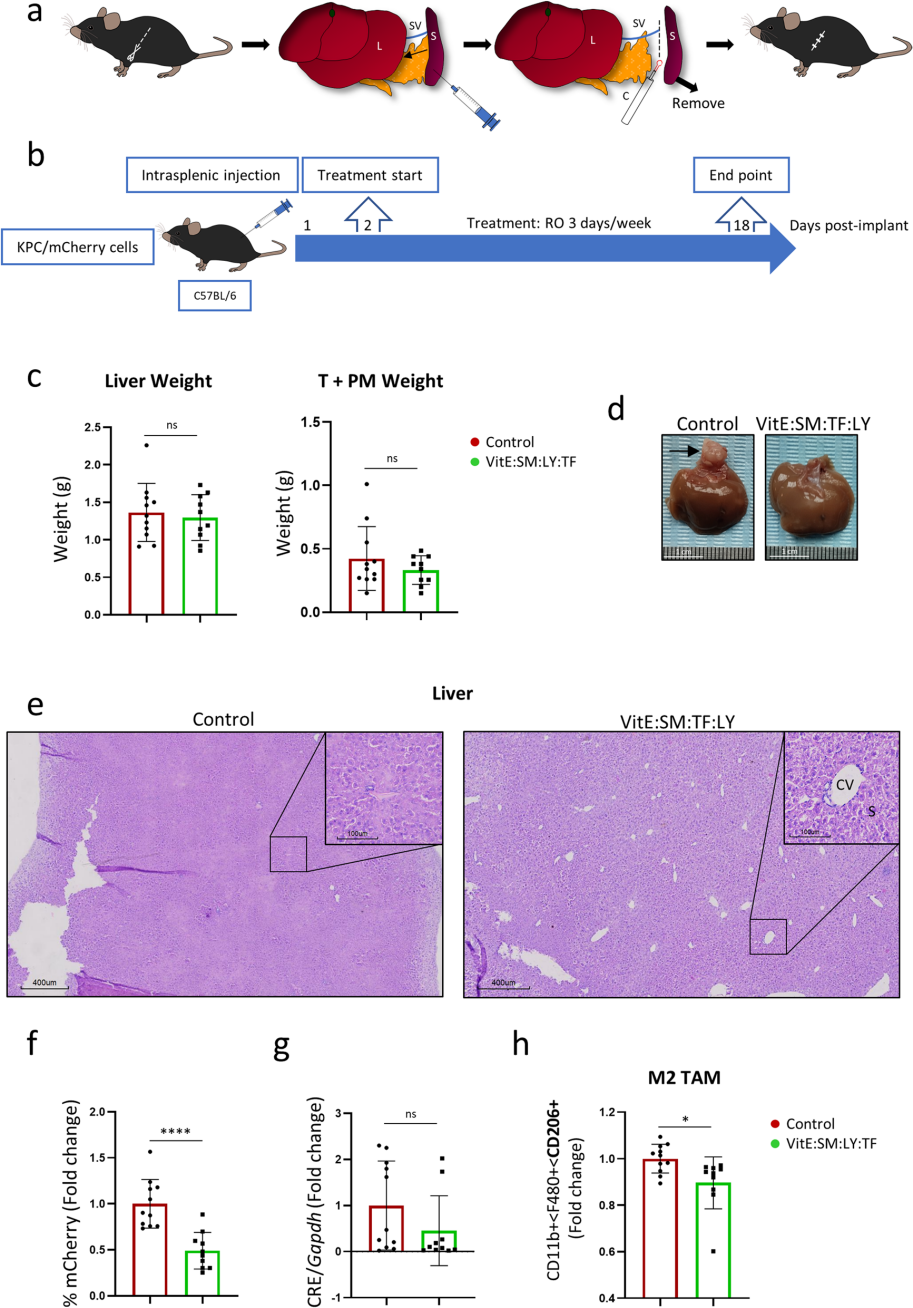
TGF-βR1 inhibitor (LY2157299)-loaded VitE:SM nanoemulsions diminish liver metastasis in an intrasplenic KPC metastasis model. **a** Schematic of the liver metastasis model involving the injection of KPC/mCherry cells into the spleen before organ resection using a cauterizer (C). *L* Liver. *S*  Spleen. *SV* Splenic Vein. **b** Schematic representation of the intervention schedules in the liver metastasis model experiment. Spleens were injected with 25,000 KPC/mCherry cells, and retro-orbital VitE:SM nanoemulsions treatments were initiated on day 2 post-intrasplenic injection and continued for 16 days, administering three doses (25 mg/kg) per week. **c**) Weight of the liver (left) or Tumor (T) + adjacent PM (right) ± SD. Data were collected from two independent experiments; Control (n = 11) and VitE:SM:LY:TF (n = 10). **d** Representative macroscopic liver images from **c**. An arrow marks the metastasis in the liver. Scale = 1cm. **e** Representative H&E staining from samples obtained from the right lobe of the liver from **d**. Scales = 400µm. Zoom areas are depicted within squares. Scales = 100 µm. *CV*  Central vein, *S*   Sinusoids. **f** Percentage of KPC/mCherry cells infiltrated in the liver. Bars represent the mean fold change ± SD, with Control set as 1.0. **g** Densitometric analysis of CRE/*Gapdh* PCR from Figure S8. Murine *Gapdh* was used as a housekeeping control. Bars represent the mean fold change ± SD, with Control set as 1.0. **h** Flow cytometry analysis of the M2 TAM marker CD206 within the CD11b +  < F480 + population. Bars represent the mean fold change ± SD, with Control set as 1.0. Unpaired t test ∗  = p < 0.05; ∗  ∗  = p < 0.01; ∗  ∗  ∗  = p < 0.001; ∗  ∗  ∗  ∗  = p < 0.0001; *ns*  not significant

KPC cells were injected into the spleens of C57Bl/6 mice, after which cauterization and removal were performed to prevent the formation of primary tumors in the injected organ (Fig. 8a). For these experiments, KPC/mCherry cells were used to track tumor cells in the different analyzed tissues. Retro-orbital administration of VitE:SM:LY:TF nanoemulsions (25 mg/kg) commenced on day 2 post-intrasplenic injection and was maintained 3 days per week until the experiment's endpoint on day 18 post-intrasplenic injection (Fig. 8b). Upon euthanasia, we observed no differences in liver or tumor weight between the control and treated groups (Fig. 8c); however, macroscopic liver metastases were detected primarily in the control mice (Fig. 8d and Figure S9). Liver tissue is structured into distinct functional units known as lobules, featuring a hexagonal arrangement of portal triads surrounding a central vein and interconnected by sinusoids [79]. In contrast to the treated mice, histological analysis of livers from control mice revealed loss of hepatocyte microarchitecture, including a reduction in the number of central veins and sinusoids, indicating tissue disorganization promoted by tumor progression (Fig. 8e).

At the cellular level, flow cytometry analysis revealed a significantly lower proportion of KPC/mCherry cells in the livers of treated mice compared to controls (Fig. 8f). Additionally, PCR analysis was performed to quantify CRE DNA copies (derived from KPC cells) in the liver, showing a reduction in amplification levels in the livers of treated mice compared to control mice (Fig. 8g and S10a). Analyzing the CD206 + TAM population in the liver by flow cytometry (CD11b + /F4/80 + /CD206 +), we observed, consistent with previous in vivo data, that the nanosystems reached liver macrophages (Figure S10b) and reduced the CD206 + TAM population (Fig. 8h). To further investigate the immune profile following treatment, we prepared single-cell suspensions from representative paraffin-embedded livers for FACS analysis of different immune populations, including M1/M0 macrophages and T cells. After treatment, we observed again a reduction in CD206 + M2 macrophages, compensated by an increase in M0/M1 macrophages (Figure S10c). Additionally, there was an increase in the overall myeloid population and CD8 + T cells (Figure S10c), aligning with the proinflammatory cytokine profile seen in the livers of treated mice (Figure S7e). Neutrophils, on the other hand, remained unchanged by the treatment (Figure S10c).

With this approximation, we successfully validated our hypothesis that reprogramming liver-infiltrating TAMs with the proposed lipid nanosystems has the potential to delay liver metastasis in PDAC. As previously mentioned, even in cases where the primary tumor is effectively resected, approximately 75% of patients experience metastatic relapse within five years post-surgical resection [8–10]. Thus, our therapeutic approach holds promise in preventing liver metastasis relapse for patients with resectable primary PDAC tumors, and could potentially be used in combination with chemotherapeutic interventions in patients with metastatic disease. The latter, however, still needs to be tested in experimental models.

**Ex vivo treatment of human 3D tumor/CAF/macrophage spheroids with VitE:SM nanoemulsions validates the translatability of the nanosystem therapy to humans.** Finally, we aimed to ascertain the translatability of our therapy to the human setting. To achieve this goal, we initially isolated and cultured monocyte-derived human macrophages. Subsequently, we induced M2 polarization using MCSF and then exposed the cells to different concentrations of VitE:SM nanoemulsions to evaluate their effect on M2 macrophage reprogramming. Using the immunosuppressive human TAM markers CD206 and CD163, we observed that VitE:SM nanoemulsions were also effective in reducing the M2 population in vitro (Figure S11a).

**Fig. 9:**
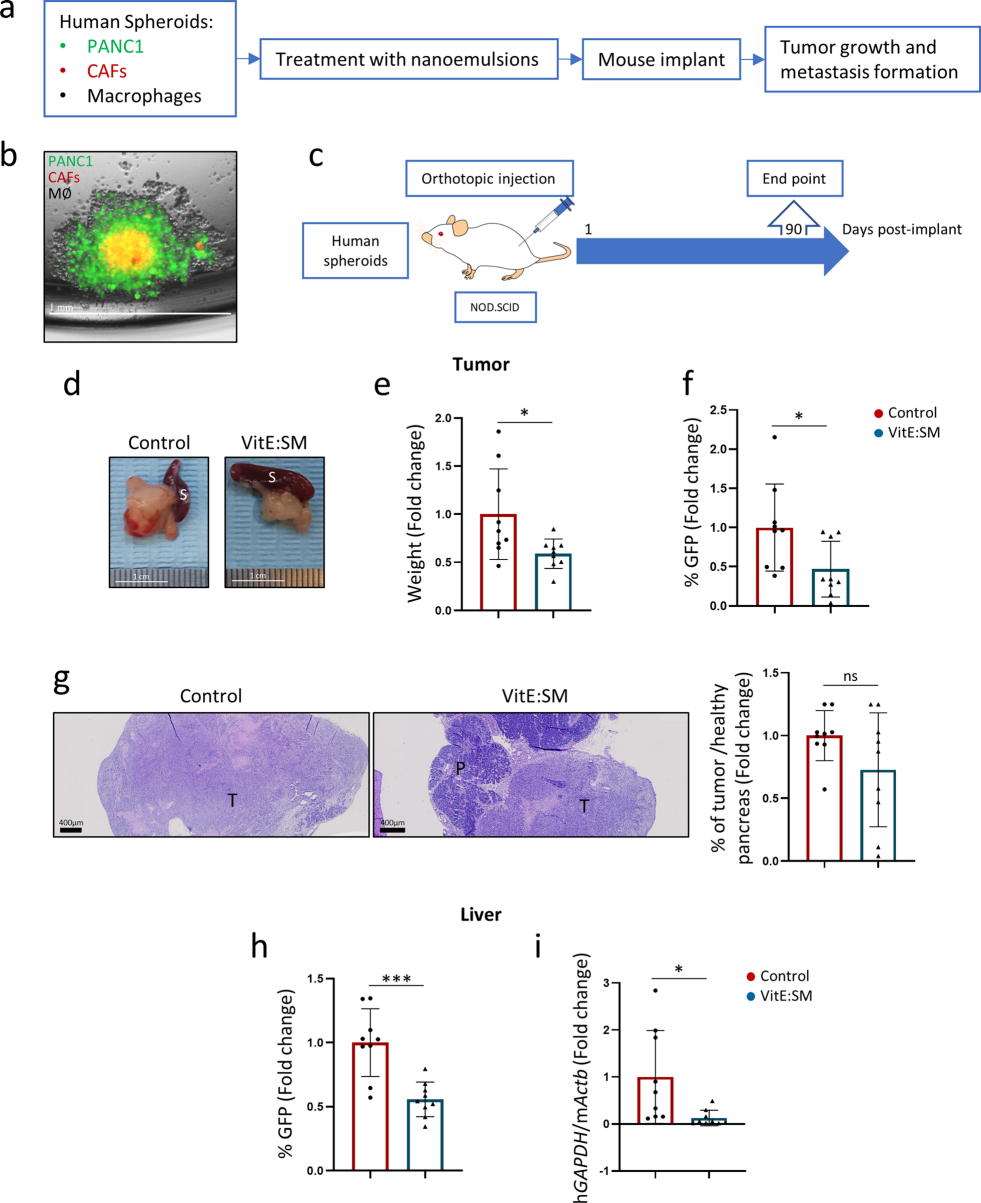
Ex vivo treatment of human 3D tumor/CAF/macrophage spheroids with VitE:SM nanoemulsions. **a** Schematic of human 3D tumor/CAF/macrophage spheroid generation, illustrating the process of generating human spheroids, comprising three populations of human cells: pancreatic cancer cells (PANC1), cancer-associated fibroblasts (CAFs) and macrophages. The 3D model was created as follows: on day one, PANC1/GFP and CAFs/mCherry were seeded in wells of a non-adherent plate, and on day 3, macrophages were added. Spheroids were treated with VitE:SM nanoemulsions (0.5 mg/mL) on day 4, the medium was changed on day 6, and on day 7, the spheroids were disaggregated and implanted orthotopically in the NOD.SCID mice. Mice were monitored for 90 days. **b** Representative fluorescence microscopy image of a human spheroid co-culture. Scale = 1mm. **c** Schematic representation of the in vivo orthotopic human spheroid model in NOD.SCID mice. Mice were euthanized at 90 days post-implantation. **d** Representative macroscopic photos of the tumors at the experimental end point (90 days post-implantation). S = Spleen served as an anatomical reference for the pancreas. Scale = 1cm. **e** Bar graph ± SD of tumor weight from **d**. Data compiled from two independent experiments (n = 2) were normalized, and the control was set as 1.0. Unpaired t test. ∗  = p < 0.05. **f** Percentage of GFP + cells (PANC1/GFP) ± SD in the tumor. Data were normalized for two independent experiments (n = 2), and the control was set as 1.0. Unpaired t test. ∗  = p < 0.05. **g** Left: Representative images (40X) of H&E-stained tumor samples from **d** are shown. *T*  Tumor, *P* Pancreas. Scale = 400 µm. Right: Ratio of the % area of tumor/healthy pancreas ± SD compared to the total histologic pancreas/tumor area quantified in the H&E slides from the left panel. Unpaired t test. *ns* not-significant. **h** Percentage of GFP + cells (PANC1/GFP) infiltrated in the liver. Bars represent the mean fold change ± SD, with Control set as 1.0. Unpaired t test. ∗  ∗  ∗  = p < 0.001. .**i** qRT-PCR analysis of the ratio of the human *GAPDH* gene/murine beta actin (m*Actb*). Bars represent the mean fold change ± SD, (n = 2 independent experiments), with Control set as 1.0. Unpaired t test. ∗  = p < 0.05

Building upon this preliminary result, we used an ex vivo 3D human spheroid cell model [36] to test the efficacy of our lipid nanoemulsions. In this scenario, empty VitE:SM nanoemulsions (without LY) were used since no differences in TAM reprogramming were observed between VitE:SM or VitE:SM:LY (LY-loaded nanoemulsions) in vitro (Fig. 6b-d). Specifically, we generated in vitro spheroids containing human pancreatic cancer cells (PANC1), human CAFs, and human macrophages to recreate the PDAC TME in culture. These spheroids were treated with VitE:SM nanoemulsions and orthotopically implanted into the pancreas of NOD.SCID mice to assess their growth and metastasis formation (Fig. 9a). To facilitate their visualization and identification in vivo, PANC1 cells were infected with a GFP-encoding lentivirus, and CAFs with an mCherry-encoding lentivirus. Fluorescence microscopy revealed the spheroid structure, with CAFs/mCherry forming a core surrounded by PANC1/GFP cells, and non-stained macrophages preferentially localizing on the exterior of the spheroid (Fig. 9b).

Established spheroids were either treated (or not) with 1 mg/mL of VitE:SM nanoemulsions, the media was changed 48 h later, and orthotopic implantation of disaggregated spheres was performed the next day. Implanted NOD.SCID mice were monitored until day 90 post-implantation (Fig. 9c). Upon euthanasia, we observed a reduction in the primary tumor burden in mice implanted with VitE:SM treated compared to untreated spheroids (Fig. 9d and Figure S11b), which we confirmed at the level of pancreatic/tumor weight (Fig. 9e). Flow cytometry analysis of cells extracted from digested tumors also revealed a reduction in the percentage of PANC1/GFP cells in tumors generated from treated spheroids (Fig. 9f). Histological analysis of these tumors showed a higher proportion of tumoral area in control tumors versus tumors generated from treated spheroids, where areas of healthy pancreas were clearly visible (Fig. 9g). At the level of metastases, we did not detect macroscopic liver metastases in either control or treated mice (Figure S11c). However, at the cellular level, we found that mice implanted with treated spheroids had a significant reduction in PANC1/GFP cells in the liver compared to controls, determined by flow cytometry (Fig. 9h). To confirm this result, we performed qRT-PCR for the human *GAPDH* gene to determine the presence of cells of human origin in the liver. In line with our flow cytometric analyses, we detected lower amplification of *GAPDH* in mice implanted with treated spheroids compared to mice implanted with non-treated spheroids (Fig. 9i).

To validate that the effects observed were at the macrophage level, a parallel experiment excluding macrophages from the PANC1/GFP:CAFs/mCherry spheroids was performed. Spheroids devoid of macrophages were treated with VitE:SM nanoemulsions under the same conditions as the previous experiment, orthotopically implanted in NOD.SCID mice, and euthanized on day 90 post-implantation (Figure S12a). Upon analysis, we observed no significant reduction in tumor weight (Figure S12b-c) nor in the level of liver metastasis. Importantly, the analysis of the liver revealed no significant decrease in the percentage of PANC1/GFP cells nor in the level of human *GAPDH* between the two experimental groups (Figure S12d-e). As such, and in line with the final conclusions determined in the murine system, these findings support the main conclusion that the effectiveness of the nanoemulsions therapy is dependent on targeting/repolarizing macrophages.

It is important to point out that the human model utilized herein has its limitations. Firstly, immunocompromised NOD.SCID mice, which have impaired macrophage function, were utilized for the engraftment of human cells in vivo. Secondly, human macrophages were pre-treated prior to injection. Thirdly, mice were not treated with the nanosystems post orthotopic implantation. Thus, while the approach does not faithfully recapitulate the metastatic setting/cascade nor a therapeutic intervention approach, these findings do offer promising preliminary support for the possible translatability of a VitE:SM-based lipid nanoemulsions therapy for human PDAC. Moreover, the results again highlight the pivotal role of macrophages as the mediators of the therapeutic effect of this nanosystem. While a fully humanized mouse model with human macrophages would have been a more ideal in vivo model, the demonstrated effectiveness of our lipid nanosystem therapy in this model of human pancreatic cancer reinforces the concept that a novel nanosystem, which also functions as a drug delivery system, holds the potential to reduce tumor growth and prevent liver metastasis for PDAC patients.

## Conclusions

In summary, we have validated a new type of nanoparticle therapy, a synergistic VitE:SM nanoparticle composition, that offers the possibility of delivering chemotherapeutics like the TGF-βR1 inhibitor, LY2157299, with the objective of reprogramming TAMs of the tumor microenvironment to prevent liver metastasis in PDAC. Both in vitro and in vivo models have demonstrated that VitE:SM nanoemulsion treatment modifies TAMs to an M0/M1 state, resulting in an immunostimulant effect that promotes the reduction of PDAC primary tumor growth and liver metastasis. The translatability of these results to the human model holds great promise for reinforcing this platform's potential use in the treatment of PDAC and potentially other metastatic cancers like colorectal cancer.

## Supplementary Information

Additional file 1

## Data Availability

FASTQ RNAseq data files generated have been deposited in Sequence Read Archive (SRA) of the National Center for Biotechnology Information (NCBI) with accession number ID: PRJNA1099468. All data underlying the results are available as part of the article. Additional information will be available on request.

## References

[1] Siegel RL, Miller KD, Wagle NS, Jemal A. Cancer statistics, 2023. CA Cancer J Clin. 2023;73(1):17–48. 36633525 10.3322/caac.21763

[2] Maitra A, Hruban RH. Pancreatic cancer. Annu Rev Pathol. 2008;3:157–88. 18039136 10.1146/annurev.pathmechdis.3.121806.154305 PMC2666336

[3] Mizrahi JD, Surana R, Valle JW, Shroff RT. Pancreatic cancer. Lancet. 2020;395(10242):2008–20. 32593337 10.1016/S0140-6736(20)30974-0

[4] https://www.cancer.net/cancer-types/pancreatic-cancer/statistics#:~:text=The%20general%205%2Dyear%20survival,disease%20when%20it%20is%20diagnosed.

[5] Deshwar AB, Sugar E, Torto D, De Jesus-Acosta A, Weiss MJ, Wolfgang CL, Le D, He J, Burkhart R, Zheng L, Laheru D, Yarchoan M. Diagnostic intervals and pancreatic ductal adenocarcinoma (PDAC) resectability: a single-center retrospective analysis. Ann Pancreat Cancer. 2018. 10.21037/apc.2018.02.01. 29683142 10.21037/apc.2018.02.01 PMC5909699

[6] Niederhuber JE, Brennan MF, Menck HR. The national cancer data base report on pancreatic cancer. Cancer. 1995;76(9):1671–7. 8635074 10.1002/1097-0142(19951101)76:9<1671::aid-cncr2820760926>3.0.co;2-r

[7] Paulson AS, Tran Cao HS, Tempero MA, Lowy AM. Therapeutic advances in pancreatic cancer. Gastroenterology. 2013;144(6):1316–26. 23622141 10.1053/j.gastro.2013.01.078

[8] Chu LC, Goggins MG, Fishman EK. Diagnosis and detection of pancreatic cancer. Cancer J. 2017;23(6):333–42. 29189329 10.1097/PPO.0000000000000290

[9] McGuigan A, Kelly P, Turkington RC, Jones C, Coleman HG, McCain RS. Pancreatic cancer: a review of clinical diagnosis, epidemiology, treatment and outcomes. World J Gastroenterol. 2018;24(43):4846–61. 30487695 10.3748/wjg.v24.i43.4846 PMC6250924

[10] Tummers WS, Groen JV, Sibinga Mulder BG, Farina-Sarasqueta A, Morreau J, Putter H, van de Velde CJ, Vahrmeijer AL, Bonsing BA, Mieog JS, Swijnenburg RJ. Impact of resection margin status on recurrence and survival in pancreatic cancer surgery. Br J Surg. 2019;106(8):1055–65. 30883699 10.1002/bjs.11115 PMC6617755

[11] Rhim AD, Mirek ET, Aiello NM, Maitra A, Bailey JM, McAllister F, Reichert M, Beatty GL, Rustgi AK, Vonderheide RH, Leach SD, Stanger BZ. EMT and dissemination precede pancreatic tumor formation. Cell. 2012;148(1–2):349–61. 22265420 10.1016/j.cell.2011.11.025 PMC3266542

[12] Yachida S, White CM, Naito Y, Zhong Y, Brosnan JA, Macgregor-Das AM, Morgan RA, Saunders T, Laheru DA, Herman JM, Hruban RH, Klein AP, Jones S, Velculescu V, Wolfgang CL, Iacobuzio-Donahue CA. Clinical significance of the genetic landscape of pancreatic cancer and implications for identification of potential long-term survivors. Clin Cancer Res. 2012;18(22):6339–47. 22991414 10.1158/1078-0432.CCR-12-1215 PMC3500447

[13] Jin T, Dai C, Xu F. Surgical and local treatment of hepatic metastasis in pancreatic ductal adenocarcinoma: recent advances and future prospects. Ther Adv Med Oncol. 2020;12:1758835920933034. 32636941 10.1177/1758835920933034 PMC7313332

[14] Pausch TM, Liu X, Cui J, Wei J, Miao Y, Heger U, Probst P, Heap S, Hackert T. Survival benefit of resection surgery for pancreatic ductal adenocarcinoma with liver metastases: a propensity score-matched seer database analysis. Cancers (Basel). 2021. 10.3390/cancers14010057. 35008223 10.3390/cancers14010057 PMC8750488

[15] Costa-Silva B, Aiello NM, Ocean AJ, Singh S, Zhang H, Thakur BK, Becker A, Hoshino A, Mark MT, Molina H, Xiang J, Zhang T, Theilen TM, García-Santos G, Williams C, Ararso Y, Huang Y, Rodrigues G, Shen TL, Labori KJ, Lothe IM, Kure EH, Hernandez J, Doussot A, Ebbesen SH, Grandgenett PM, Hollingsworth MA, Jain M, Mallya K, Batra SK, Jarnagin WR, Schwartz RE, Matei I, Peinado H, Stanger BZ, Bromberg J, Lyden D. Pancreatic cancer exosomes initiate pre-metastatic niche formation in the liver. Nat Cell Biol. 2015;17(6):816–26. 25985394 10.1038/ncb3169 PMC5769922

[16] Ligorio M, Sil S, Malagon-Lopez J, Nieman LT, Misale S, Di Pilato M, Ebright RY, Karabacak MN, Kulkarni AS, Liu A, Vincent Jordan N, Franses JW, Philipp J, Kreuzer J, Desai N, Arora KS, Rajurkar M, Horwitz E, Neyaz A, Tai E, Magnus NKC, Vo KD, Yashaswini CN, Marangoni F, Boukhali M, Fatherree JP, Damon LJ, Xega K, Desai R, Choz M, Bersani F, Langenbucher A, Thapar V, Morris R, Wellner UF, Schilling O, Lawrence MS, Liss AS, Rivera MN, Deshpande V, Benes CH, Maheswaran S, Haber DA, Fernandez-Del-Castillo C, Ferrone CR, Haas W, Aryee MJ, Ting DT. Stromal microenvironment shapes the intratumoral architecture of pancreatic cancer. Cell. 2019;178(1):160-175.e27. 31155233 10.1016/j.cell.2019.05.012 PMC6697165

[17] Marvin DL, Heijboer R, Ten Dijke P, Ritsma L. TGF-β signaling in liver metastasis. Clin Transl Med. 2020;10(7): e160. 33252863 10.1002/ctm2.160 PMC7701955

[18] Gil-Bernabé AM, Ferjancic S, Tlalka M, Zhao L, Allen PD, Im JH, Watson K, Hill SA, Amirkhosravi A, Francis JL, Pollard JW, Ruf W, Muschel RJ. Recruitment of monocytes/macrophages by tissue factor-mediated coagulation is essential for metastatic cell survival and premetastatic niche establishment in mice. Blood. 2012;119(13):3164–75. 22327225 10.1182/blood-2011-08-376426

[19] Labelle M, Begum S, Hynes RO. Platelets guide the formation of early metastatic niches. Proc Natl Acad Sci U S A. 2014;111(30):E3053–61. 25024172 10.1073/pnas.1411082111 PMC4121772

[20] Peinado H, Zhang H, Matei IR, Costa-Silva B, Hoshino A, Rodrigues G, Psaila B, Kaplan RN, Bromberg JF, Kang Y, Bissell MJ, Cox TR, Giaccia AJ, Erler JT, Hiratsuka S, Ghajar CM, Lyden D. Pre-metastatic niches: organ-specific homes for metastases. Nat Rev Cancer. 2017;17(5):302–17. 28303905 10.1038/nrc.2017.6

[21] Ho WJ, Jaffee EM, Zheng L. The tumour microenvironment in pancreatic cancer - clinical challenges and opportunities. Nat Rev Clin Oncol. 2020;17(9):527–40. 32398706 10.1038/s41571-020-0363-5 PMC7442729

[22] Apte MV, Wilson JS, Lugea A, Pandol SJ. A starring role for stellate cells in the pancreatic cancer microenvironment. Gastroenterology. 2013;144(6):1210–9. 23622130 10.1053/j.gastro.2012.11.037 PMC3729446

[23] Clark CE, Hingorani SR, Mick R, Combs C, Tuveson DA, Vonderheide RH. Dynamics of the immune reaction to pancreatic cancer from inception to invasion. Cancer Res. 2007;67(19):9518–27. 17909062 10.1158/0008-5472.CAN-07-0175

[24] Lankadasari MB, Mukhopadhyay P, Mohammed S, Harikumar KB. TAMing pancreatic cancer: combat with a double edged sword. Mol Cancer. 2019;18(1):48. 30925924 10.1186/s12943-019-0966-6 PMC6441154

[25] Thomas H. Pancreatic cancer: Infiltrating macrophages support liver metastasis. Nat Rev Gastroenterol Hepatol. 2016;13(6):313. 27118627 10.1038/nrgastro.2016.71

[26] Nielsen SR, Quaranta V, Linford A, Emeagi P, Rainer C, Santos A, Ireland L, Sakai T, Sakai K, Kim YS, Engle D, Campbell F, Palmer D, Ko JH, Tuveson DA, Hirsch E, Mielgo A, Schmid MC. Macrophage-secreted granulin supports pancreatic cancer metastasis by inducing liver fibrosis. Nat Cell Biol. 2016;18(5):549–60. 27088855 10.1038/ncb3340 PMC4894551

[27] Trombetta AC, Soldano S, Contini P, Tomatis V, Ruaro B, Paolino S, Brizzolara R, Montagna P, Sulli A, Pizzorni C, Smith V, Cutolo M. A circulating cell population showing both M1 and M2 monocyte/macrophage surface markers characterizes systemic sclerosis patients with lung involvement. Respir Res. 2018;19(1):186. 30249259 10.1186/s12931-018-0891-z PMC6154930

[28] Gordon S. Alternative activation of macrophages. Nat Rev Immunol. 2003;3(1):23–35. 12511873 10.1038/nri978

[29] Kurahara H, Shinchi H, Mataki Y, Maemura K, Noma H, Kubo F, Sakoda M, Ueno S, Natsugoe S, Takao S. Significance of M2-polarized tumor-associated macrophage in pancreatic cancer. J Surg Res. 2011;167(2):e211–9. 19765725 10.1016/j.jss.2009.05.026

[30] Alonso-Nocelo M, Ruiz-Cañas L, Sancho P, Görgülü K, Alcalá S, Pedrero C, Vallespinos M, López-Gil JC, Ochando M, García-García E, David Trabulo SM, Martinelli P, Sánchez-Tomero P, Sánchez-Palomo C, Gonzalez-Santamaría P, Yuste L, Wörmann SM, Kabacaoğlu D, Earl J, Martin A, Salvador F, Valle S, Martin-Hijano L, Carrato A, Erkan M, García-Bermejo L, Hermann PC, Algül H, Moreno-Bueno G, Heeschen C, Portillo F, Cano A, Sainz B Jr. Macrophages direct cancer cells through a LOXL2-mediated metastatic cascade in pancreatic ductal adenocarcinoma. Gut. 2023;72(2):345–59. 35428659 10.1136/gutjnl-2021-325564 PMC9872246

[31] Griesmann H, Drexel C, Milosevic N, Sipos B, Rosendahl J, Gress TM, Michl P. Pharmacological macrophage inhibition decreases metastasis formation in a genetic model of pancreatic cancer. Gut. 2017;66(7):1278–85. 27013602 10.1136/gutjnl-2015-310049

[32] Khan SU, Khan MU, Din AU, M., Khan, I. M., Khan, M. I., Bungau, S., Hassan, S. S. U. Reprogramming tumor-associated macrophages as a unique approach to target tumor immunotherapy. Front Immunol. 2023. 10.3389/fimmu.2023.1166487. 37138860 10.3389/fimmu.2023.1166487 PMC10149956

[33] Li S-L, Hou H-Y, Chu X, Zhu Y-Y, Zhang Y-J, Duan M-D, Liu J, Liu Y. Nanomaterials-involved tumor-associated macrophages’ reprogramming for antitumor therapy. ACS Nano. 2024;18(11):7769–95. 38420949 10.1021/acsnano.3c12387

[34] Bouzo BL, Calvelo M, Martín-Pastor M, García-Fandiño R, de la Fuente M. In vitro-in silico modeling approach to rationally designed simple and versatile drug delivery systems. J Phys Chem B. 2020;124(28):5788–800. 32525313 10.1021/acs.jpcb.0c02731

[35] Bouzo BL, Lores S, Jatal R, Alijas S, Alonso MJ, Conejos-Sánchez I, de la Fuente M. Sphingomyelin nanosystems loaded with uroguanylin and etoposide for treating metastatic colorectal cancer. Sci Rep. 2021;11(1):17213. 34446776 10.1038/s41598-021-96578-z PMC8390746

[36] Bidan N, Lores S, Vanhecke A, Nicolas V, Domenichini S, López R, de la Fuente M, Mura S. Before in vivo studies: In vitro screening of sphingomyelin nanosystems using a relevant 3D multicellular pancreatic tumor spheroid model. Int J Pharm. 2022;617: 121577. 35167901 10.1016/j.ijpharm.2022.121577

[37] Jatal R, Mendes Saraiva S, Vázquez-Vázquez C, Lelievre E, Coqueret O, López-López R, de la Fuente M. Sphingomyelin nanosystems decorated with TSP-1 derived peptide targeting senescent cells. Int J Pharm. 2022;617: 121618. 35219823 10.1016/j.ijpharm.2022.121618

[38] Nagachinta S, Becker G, Dammicco S, Serrano ME, Leroi N, Bahri MA, Plenevaux A, Lemaire C, Lopez R, Luxen A, de la Fuente M. Radiolabelling of lipid-based nanocarriers with fluorine-18 for in vivo tracking by PET. Colloids Surf B Biointerfaces. 2020;188: 110793. 31982792 10.1016/j.colsurfb.2020.110793

[39] Díez-Villares S, Pellico J, Gómez-Lado N, Grijalvo S, Alijas S, Eritja R, Herranz F, Aguiar P, de la Fuente M. Biodistribution of (68/67)Ga-Radiolabeled Sphingolipid Nanoemulsions by PET and SPECT Imaging. Int J Nanomedicine. 2021;16:5923–35. 34475757 10.2147/IJN.S316767 PMC8405882

[40] Asmis R, Jelk J. Vitamin E supplementation of human macrophages prevents neither foam cell formation nor increased susceptibility of foam cells to lysis by oxidized LDL. Arterioscler Thromb Vasc Biol. 2000;20(9):2078–86. 10978252 10.1161/01.atv.20.9.2078

[41] Lee GY, Han SN. The Role of Vitamin E in Immunity. Nutrients. 2018. 10.3390/nu10111614. 30388871 10.3390/nu10111614 PMC6266234

[42] Beharka AA, Wu D, Serafini M, Meydani SN. Mechanism of vitamin E inhibition of cyclooxygenase activity in macrophages from old mice: role of peroxynitrite. Free Radic Biol Med. 2002;32(6):503–11. 11958951 10.1016/s0891-5849(01)00817-6

[43] Asbaghi O, Sadeghian M, Nazarian B, Sarreshtedari M, Mozaffari-Khosravi H, Maleki V, Alizadeh M, Shokri A, Sadeghi O. The effect of vitamin E supplementation on selected inflammatory biomarkers in adults: a systematic review and meta-analysis of randomized clinical trials. Sci Rep. 2020;10(1):17234. 33057114 10.1038/s41598-020-73741-6 PMC7560744

[44] Hannun YA, Obeid LM. Sphingolipids and their metabolism in physiology and disease. Nat Rev Mol Cell Biol. 2018;19(3):175–91. 29165427 10.1038/nrm.2017.107 PMC5902181

[45] Maceyka M, Spiegel S. Sphingolipid metabolites in inflammatory disease. Nature. 2014;510(7503):58–67. 24899305 10.1038/nature13475 PMC4320971

[46] Sakamoto H, Yoshida T, Sanaki T, Shigaki S, Morita H, Oyama M, Mitsui M, Tanaka Y, Nakano T, Mitsutake S, Igarashi Y, Takemoto H. Possible roles of long-chain sphingomyelines and sphingomyelin synthase 2 in mouse macrophage inflammatory response. Biochem Biophys Res Commun. 2017;482(2):202–7. 27836537 10.1016/j.bbrc.2016.11.041

[47] Camell CD, Nguyen KY, Jurczak MJ, Christian BE, Shulman GI, Shadel GS, Dixit VD. Macrophage-specific de Novo synthesis of ceramide is dispensable for inflammasome-driven inflammation and insulin resistance in obesity. J Biol Chem. 2015;290(49):29402–13. 26438821 10.1074/jbc.M115.680199 PMC4705943

[48] Chiorean EG, Picozzi V, Li CP, Peeters M, Maurel J, Singh J, Golan T, Blanc JF, Chapman SC, Hussain AM, Johnston EL, Hochster HS. Efficacy and safety of abemaciclib alone and with PI3K/mTOR inhibitor LY3023414 or galunisertib versus chemotherapy in previously treated metastatic pancreatic adenocarcinoma: a randomized controlled trial. Cancer Med. 2023;12(20):20353–64. 37840530 10.1002/cam4.6621 PMC10652308

[49] Melisi D, Garcia-Carbonero R, Macarulla T, Pezet D, Deplanque G, Fuchs M, Trojan J, Oettle H, Kozloff M, Cleverly A, Smith C, Estrem ST, Gueorguieva I, Lahn MMF, Blunt A, Benhadji KA, Tabernero J. Galunisertib plus gemcitabine vs. gemcitabine for first-line treatment of patients with unresectable pancreatic cancer. Br J Cancer. 2018. 10.1038/s41416-018-0246-z. 30318515 10.1038/s41416-018-0246-z PMC6251034

[50] Melisi D, Oh DY, Hollebecque A, Calvo E, Varghese A, Borazanci E, Macarulla T, Merz V, Zecchetto C, Zhao Y, Gueorguieva I, Man M, Gandhi L, Estrem ST, Benhadji KA, Lanasa MC, Avsar E, Guba SC, Garcia-Carbonero R. Safety and activity of the TGFβ receptor I kinase inhibitor galunisertib plus the anti-PD-L1 antibody durvalumab in metastatic pancreatic cancer. J Immunother Cancer. 2021. 10.1136/jitc-2020-002068. 33688022 10.1136/jitc-2020-002068 PMC7944986

[51] Díaz-Alejo JF, April-Monn S, Cihova M, Buocikova V, Villalón López J, Urbanova M, Lechuga CG, Tomas M, Dubovan P, Sánchez BL, Páez SC, Sanjuanbenito A, Lobo E, Romio de la Heras E, Guerra C, de la Pinta C, Barreto Melian E, Rodríguez Garrote M, Carrato A, Ruiz-Cañas L, Sainz B Jr, Torres A, Smolkova B, Earl J. Establishment of pancreatic cancer-derived tumor organoids and fibroblasts from fresh tissue. J Vis Exp. 2023. 10.3791/65229. 37306424 10.3791/65229

[52] Valle S, Alcalá S, Martin-Hijano L, Cabezas-Sáinz P, Navarro D, Muñoz ER, Yuste L, Tiwary K, Walter K, Ruiz-Cañas L, Alonso-Nocelo M, Rubiolo JA, González-Arnay E, Heeschen C, Garcia-Bermejo L, Hermann PC, Sánchez L, Sancho P, Fernández-Moreno M, Sainz B, Jr.,. Exploiting oxidative phosphorylation to promote the stem and immunoevasive properties of pancreatic cancer stem cells. Nat Commun. 2020;11(1):5265. 33067432 10.1038/s41467-020-18954-z PMC7567808

[53] Chomczynski P, Sacchi N. Single-step method of RNA isolation by acid guanidinium thiocyanate-phenol-chloroform extraction. Anal Biochem. 1987;162(1):156–9. 2440339 10.1006/abio.1987.9999

[54] Alcalá S, Villarino L, Ruiz-Cañas L, Couceiro JR, Martínez-Calvo M, Palencia-Campos A, Navarro D, Cabezas-Sainz P, Rodriguez-Arabaolaza I, Cordero-Barreal A, Trilla-Fuertes L, Rubiolo JA, Batres-Ramos S, Vallespinos M, González-Páramos C, Rodríguez J, Gámez-Pozo A, Vara JÁF, Fernández SF, Berlinches AB, Moreno-Mata N, Redondo AMT, Carrato A, Hermann PC, Sánchez L, Torrente S, Fernández-Moreno MÁ, Mascareñas JL, Sainz B. Targeting cancer stem cell OXPHOS with tailored ruthenium complexes as a new anti-cancer strategy. J Exp Clin Cancer Res. 2024;43(1):33. 38281027 10.1186/s13046-023-02931-7 PMC10821268

[55] O’Brien M, Ernst M, Poh AR. An intrasplenic injection model of pancreatic cancer metastasis to the liver in mice. STAR Protoc. 2023;4(1): 102021. 36638017 10.1016/j.xpro.2022.102021 PMC9846119

[56] Sainz B Jr, Alcala S, Garcia E, Sanchez-Ripoll Y, Azevedo MM, Cioffi M, Tatari M, Miranda-Lorenzo I, Hidalgo M, Gomez-Lopez G, Cañamero M, Erkan M, Kleeff J, García-Silva S, Sancho P, Hermann PC, Heeschen C. Microenvironmental hCAP-18/LL-37 promotes pancreatic ductal adenocarcinoma by activating its cancer stem cell compartment. Gut. 2015;64(12):1921–35. 25841238 10.1136/gutjnl-2014-308935

[57] Bidan N, Dunsmore G, Ugrinic M, Bied M, Moreira M, Deloménie C, Ginhoux F, Blériot C, de la Fuente M, Mura S. Multicellular tumor spheroid model to study the multifaceted role of tumor-associated macrophages in PDAC. Drug Deliv Transl Res. 2024;14(8):2085–99. 38062286 10.1007/s13346-023-01479-5

[58] Qiao X, Hu Z, Xiong F, Yang Y, Peng C, Wang D, Li X. Lipid metabolism reprogramming in tumor-associated macrophages and implications for therapy. Lipids Health Dis. 2023;22(1):45. 37004014 10.1186/s12944-023-01807-1 PMC10064535

[59] Kuninty PR, Binnemars-Postma K, Jarray A, Pednekar KP, Heinrich MA, Pijffers HJ, ten Hoopen H, Storm G, van Hoogevest P, den Otter WK, Prakash J. Cancer immune therapy using engineered ‛tail-flipping’ nanoliposomes targeting alternatively activated macrophages. Nat Commun. 2022;13(1):4548. 35927238 10.1038/s41467-022-32091-9 PMC9352736

[60] Tabraue C, Lara PC, De Mirecki-Garrido M, De La Rosa JV, López-Blanco F, Fernández-Pérez L, Boscá L, Castrillo A. LXR signaling regulates macrophage survival and inflammation in response to ionizing radiation. Int J Radiat Oncol Biol Phys. 2019;104(4):913–23. 30922944 10.1016/j.ijrobp.2019.03.028

[61] Gordon S, Martinez FO. Alternative activation of macrophages: mechanism and functions. Immunity. 2010;32(5):593–604. 20510870 10.1016/j.immuni.2010.05.007

[62] Murray PJ, Wynn TA. Protective and pathogenic functions of macrophage subsets. Nat Rev Immunol. 2011;11(11):723–37. 21997792 10.1038/nri3073 PMC3422549

[63] Lumeng CN, Saltiel AR. Inflammatory links between obesity and metabolic disease. J Clin Invest. 2011;121(6):2111–7. 21633179 10.1172/JCI57132 PMC3104776

[64] Menjivar RE, Nwosu ZC, Du W, Donahue KL, Hong HS, Espinoza C, Brown K, Velez-Delgado A, Yan W, Lima F, Bischoff A, Kadiyala P, Salas-Escabillas D, Crawford HC, Bednar F, Carpenter E, Zhang Y, Halbrook CJ, Lyssiotis CA, Pasca di Magliano M. Arginase 1 is a key driver of immune suppression in pancreatic cancer. Elife. 2023. 10.7554/eLife.80721. 36727849 10.7554/eLife.80721 PMC10260021

[65] Sun R, Gu X, Lei C, Chen L, Chu S, Xu G, Doll MA, Tan Y, Feng W, Siskind L, McClain CJ, Deng Z. Neutral ceramidase-dependent regulation of macrophage metabolism directs intestinal immune homeostasis and controls enteric infection. Cell Rep. 2022;38(13): 110560. 35354041 10.1016/j.celrep.2022.110560 PMC9007044

[66] Hwang J, Zheng M, Wiraja C, Cui M, Yang L, Xu C. Reprogramming of macrophages with macrophage cell membrane-derived nanoghosts. Nanoscale Adv. 2020;2(11):5254–62. 36132036 10.1039/d0na00572j PMC9419214

[67] Hingorani SR, Wang L, Multani AS, Combs C, Deramaudt TB, Hruban RH, Rustgi AK, Chang S, Tuveson DA. Trp53R172H and KrasG12D cooperate to promote chromosomal instability and widely metastatic pancreatic ductal adenocarcinoma in mice. Cancer Cell. 2005;7(5):469–83. 15894267 10.1016/j.ccr.2005.04.023

[68] Li Z, Ding Y, Liu J, Wang J, Mo F, Wang Y, Chen-Mayfield TJ, Sondel PM, Hong S, Hu Q. Depletion of tumor associated macrophages enhances local and systemic platelet-mediated anti-PD-1 delivery for post-surgery tumor recurrence treatment. Nat Commun. 2022;13(1):1845. 35387972 10.1038/s41467-022-29388-0 PMC8987059

[69] Lu J, Liu X, Liao YP, Salazar F, Sun B, Jiang W, Chang CH, Jiang J, Wang X, Wu AM, Meng H, Nel AE. Nano-enabled pancreas cancer immunotherapy using immunogenic cell death and reversing immunosuppression. Nat Commun. 2017;8(1):1811. 29180759 10.1038/s41467-017-01651-9 PMC5703845

[70] Lorkowski ME, Atukorale PU, Bielecki PA, Tong KH, Covarrubias G, Zhang Y, Loutrianakis G, Moon TJ, Santulli AR, Becicka WM, Karathanasis E. Immunostimulatory nanoparticle incorporating two immune agonists for the treatment of pancreatic tumors. J Control Release. 2021;330:1095–105. 33188827 10.1016/j.jconrel.2020.11.014 PMC7906920

[71] Park JE, Dutta B, Tse SW, Gupta N, Tan CF, Low JK, Yeoh KW, Kon OL, Tam JP, Sze SK. Hypoxia-induced tumor exosomes promote M2-like macrophage polarization of infiltrating myeloid cells and microRNA-mediated metabolic shift. Oncogene. 2019;38(26):5158–73. 30872795 10.1038/s41388-019-0782-x

[72] Dzik JM. Evolutionary roots of arginase expression and regulation. Front Immunol. 2014;5:544. 25426114 10.3389/fimmu.2014.00544 PMC4224125

[73] Arlauckas SP, Garren SB, Garris CS, Kohler RH, Oh J, Pittet MJ, Weissleder R. Arg1 expression defines immunosuppressive subsets of tumor-associated macrophages. Theranostics. 2018;8(21):5842–54. 30613266 10.7150/thno.26888 PMC6299430

[74] Kano MR, Bae Y, Iwata C, Morishita Y, Yashiro M, Oka M, Fujii T, Komuro A, Kiyono K, Kaminishi M, Hirakawa K, Ouchi Y, Nishiyama N, Kataoka K, Miyazono K. Improvement of cancer-targeting therapy, using nanocarriers for intractable solid tumors by inhibition of TGF-beta signaling. Proc Natl Acad Sci U S A. 2007;104(9):3460–5. 17307870 10.1073/pnas.0611660104 PMC1800736

[75] Yingling JM, Blanchard KL, Sawyer JS. Development of TGF-beta signalling inhibitors for cancer therapy. Nat Rev Drug Discov. 2004;3(12):1011–22. 15573100 10.1038/nrd1580

[76] Melisi D, Ishiyama S, Sclabas GM, Fleming JB, Xia Q, Tortora G, Abbruzzese JL, Chiao PJ. LY2109761, a novel transforming growth factor beta receptor type I and type II dual inhibitor, as a therapeutic approach to suppressing pancreatic cancer metastasis. Mol Cancer Ther. 2008;7(4):829–40. 18413796 10.1158/1535-7163.MCT-07-0337 PMC3088432

[77] He M, Henderson M, Muth S, Murphy A, Zheng L. Preclinical mouse models for immunotherapeutic and non-immunotherapeutic drug development for pancreatic ductal adenocarcinoma. Annals of Pancreatic Cancer. 2020. 10.21037/apc.2020.03.03. 32832900 10.21037/apc.2020.03.03 PMC7440242

[78] Poh AR, O’Brien M, Chisanga D, He H, Baloyan D, Traichel J, Dijkstra C, Chopin M, Nutt S, Whitehead L, Boon L, Parkin A, Lowell C, Pajic M, Shi W, Nikfarjam M, Ernst M. Inhibition of HCK in myeloid cells restricts pancreatic tumor growth and metastasis. Cell Rep. 2022;41(2): 111479. 36223746 10.1016/j.celrep.2022.111479 PMC11299506

[79] Drew J, Machesky LM. The liver metastatic niche: modelling the extracellular matrix in metastasis. Dis Model Mech. 2021. 10.1242/dmm.048801. 33973625 10.1242/dmm.048801 PMC8077555

