# Supplementary material for: Reprogramming tumor-associated macrophages with lipid nanosystems reduces PDAC tumor burden and liver metastasis
Source: J Nanobiotechnology. 2024 Dec 24;22:795. doi: 10.1186/s12951-024-03010-5 (PMC11668009; doi:10.1186/s12951-024-03010-5)
Supplement: Supplementary file 1 — Additional file 1 [file 12951_2024_3010_MOESM1_ESM.docx]

**Supporting Information**

**Reprogramming Tumor-Associated Macrophages with Lipid Nanosystems Reduces PDAC Tumor Burden and Liver Metastasis**

Adrián Palencia-Campos^1,2^, Laura Ruiz-Cañas^1,2,3^, Marcelina Abal-Sanisidro^4,5,6^, Juan Carlos López-Gil^1,2,7^, Sandra Batres-Ramos^1,2^, Sofia Mendes Saraiva^4,6,8^, Balbino Yagüe^1,2^, Diego Navarro^1,2,7^, Sonia Alcalá^1,2^, Juan A. Rubiolo^9,10^, Nadège Bidan^11^, Laura Sánchez^9^, Simona Mura^11^, Patrick C. Hermann^12^, María de la Fuente^4,6,13^, Bruno Sainz, Jr.^1,2,6,†^

^1^Cancer Stem Cells and Fibroinflammatory Microenvironment Group, Instituto de Investigaciones Biomédicas (IIBm) Sols-Morreale CSIC-UAM, 28029 Madrid, Spain.

^2^Biomarkers and Personalized Approach to Cancer Group (BIOPAC), Area 3 Cancer, Instituto Ramón y Cajal de Investigación Sanitaria (IRYCIS), 28049, Madrid, Spain.

^3^Biobanco Hospital Universitario Ramón y Cajal, IRYCIS, Madrid, Spain.

^4^Nano-Oncology and Translational Therapeutics group, IDIS, Complexo Hospitalario Universitario de Santiago de Compostela, 15706 Santiago de Compostela, Spain.

^5^University of Santiago de Compostela (USC), 15782 Santiago de Compostela, Spain.

^6^Centro de Investigación Biomédica en Red, Área Cáncer, CIBERONC, ISCIII, Madrid, Spain.

^7^Department of Biochemistry, Autónoma University of Madrid (UAM), 28029 Madrid, Spain.

^8^CPIRN-IPG- Center of Potential and Innovation of Natural Resources, Polytechnic Institute of Guarda, Av. Dr. Francisco de Sá Carneiro, No. 50, 6300-559 Guarda, Portugal.

^9^Department of Zoology, Genetics and Physical Anthropology, Faculty of Veterinary, University of Santiago de Compostela (USC), Lugo, Spain.

^10^Laboratorio Mixto de Biotecnología Acuática, Facultad de Ciencias Bioquímicas y Farmacéuticas, UNR, 2000, Rosario, Argentina.

^11^Université Paris-Saclay, CNRS, Institut Galien Paris-Saclay, 91400 Orsay, France

^12^Department of Internal Medicine I, Ulm University, Ulm, Germany

^13^DIVERSA Technologies S.L., Edificio Emprendia, Campus Sur, 15782 Santiago de Compostela, Spain.

**^†^Correspondence:** Bruno Sainz, Jr.: [bsainz@iib.uam.es](mailto:bsainz@iib.uam.es)

This section includes:

**Supplementary Tables S1-S3**

**Supplementary Figures S1-S12**

| Gene | Species | Forward Primer (5´-3´) | Reverse Primer (5´-3´) |
| --- | --- | --- | --- |
| *Actb* | Mouse | TTACGGATGTCAACGTCACAGTTC | ACTATTGGCAACGAGCGGTTC |
| *GAPDH* | Human | GAAGGTGAAGGTCGGAGTC | GAAGATGGTGATGGGATTTC |
| *Arg1* | Mouse | TCATTTGGGTGGATGCTCACAC | GAGAATCCTGGTACATCTGGGAA |
| *Egr2* | Mouse | AACGGAGTGGCGGGAGAT | ATGGGAGCGAAGCTACTCGGAT |
| *Il12b* | Mouse | TTGAACTGGCGTTGGAAGCACG | CCACCTGTGAGTTCTTCAAAGGC |
| *Cd86* | Mouse | ACGTATTGGAAGGAGATTACAGCT | TCTGTCAGCGTTACTATCCCGC |
| *Il1b* | Mouse | TGGACCTTCCAGGATGAGGACA | GTTCATCTCGGAGCCTGTAGTG |
| *Pglyrp1* | Mouse | CCGCAATGTGCAGCATTACCAC | TGGTCACCCTTGATGTTCCAGC |
| *Ccl2* | Mouse | GCTACAAGAGGATCACCAGCAG | GTCTGGACCCATTCCTTCTTGG |
| *Mpo* | Mouse | CGTGTCAAGTGGCTGTGCCTAT | AACCAGCGTACAAAGGCACGGT |
| *Il6* | Mouse | TACCACTTCACAAGTCGGAGGC | CTGCAAGTGCATCATCGTTGTTC |
| *Tnf* | Mouse | GGTGCCTATGTCTCAGCCTCTT | GCCATAGAACTGATGAGAGGGAG |
| *Ptgs2* | Mouse | GCGACATACTCAAGCAGGAGCA | AGTGGTAACCGCTCAGGTGTTG |
| *Cxcl1* | Mouse | TCCAGAGCTTGAAGGTGTTGCC | AACCAAGGGAGCTTCAGGGTCA |
| *Gzmb* | Mouse | CAGGAGAAGACCCAGCAAGTCA | CTCACAGCTCTAGTCCTCTTGG |

**Table S1. RT-qPCR primers.**

|  | Color | Ref. | Company | Dilution | Ex (nM) | Em (nM) | Channel |
| --- | --- | --- | --- | --- | --- | --- | --- |
| Murine | | | | | | | |
| CD45 | PE-Cyanine7 | 25-0451-82 | Invitrogen | 1:80 | 561 | 780/60 | YL4 |
| CD11b | PerCP-Cyanine5.5 | 65-0112 | TONBO | 1:50 | 488 | 695/40 | BL3 |
| F4/80 | APC | 130-116-547 | Miltenyi Biotec | 1:50 | 637 | 670/14 | RL1 |
| CD206 | PE | 12-2061-82 | Invitrogen eBioscience | 1:50 | 561 | 585/16 | YL1 |
| CD206 | PE-Cyanine7 | 141719 | Biolegend | 1:50 | 561 | 780/60 | YL4 |
| Ly6-G | AF700 | 127621 | Biolegend | 1.25:100 | 702 | 723 | RL2 |
| CD4 | PE | 100512 | Biolegend | 1:100 | 561 | 585/16 | YL1 |
| CD8 | APC | 100712 | Biolegend | 1:100 | 637 | 670/14 | RL1 |
| Others | | | | | | | |
| TopFluor |  |  |  |  | 488 | 530/30 | BL1 |
| mCherry |  |  |  |  | 561 | 620/15 | YL2 |
| DAPI | DAPI | 62248 | Thermo Scientific | 2µg/mL | 505 | 440/50 | VL1 |
| Human | | | | | | | |
| CD206 | FITC | 130-095-131 | Miltenyi Biotec | 1:20 | 488 | 530/30 | BL1 |
| CD163 | PE | 130-112-128 | Miltenyi Biotec | 1:50 | 561 | 585/16 | YL1 |

**Table S2. Flow cytometry antibodies.**

| Sample | Sex | Age | Weight | ALT | AST | GGT |
| --- | --- | --- | --- | --- | --- | --- |
| Mouse 1 | Male | 16 weeks | 24 g | 22 U/l (28-129 U/l) | 28 U/l (46-392 U/l) | <5 U/l (0-9 U/l) |
| Mouse 2 | Male | 16 weeks | 23 g | 20 U/l (28-129 U/l) | 19 U/l (46-392 U/l) | <5 U/l (0-9 U/l) |
| Mouse 3 | Male | 16 weeks | 23 g | 24 U/l (28-129 U/l) | 24 U/l (46-392 U/l) | <5 U/l (0-9 U/l) |

**Table S3. Liver toxicity evaluation of VitE:SM nanoemulsion treatment.** Liver function test were performed in three representative mice treated with VitE:SM nanoemulsions. Normal values references provided by Charles River Co are in parenthesis. ALT=alanine transaminase, AST=aspartate aminotransferase, GGT=gamma-glutamyl transferase.


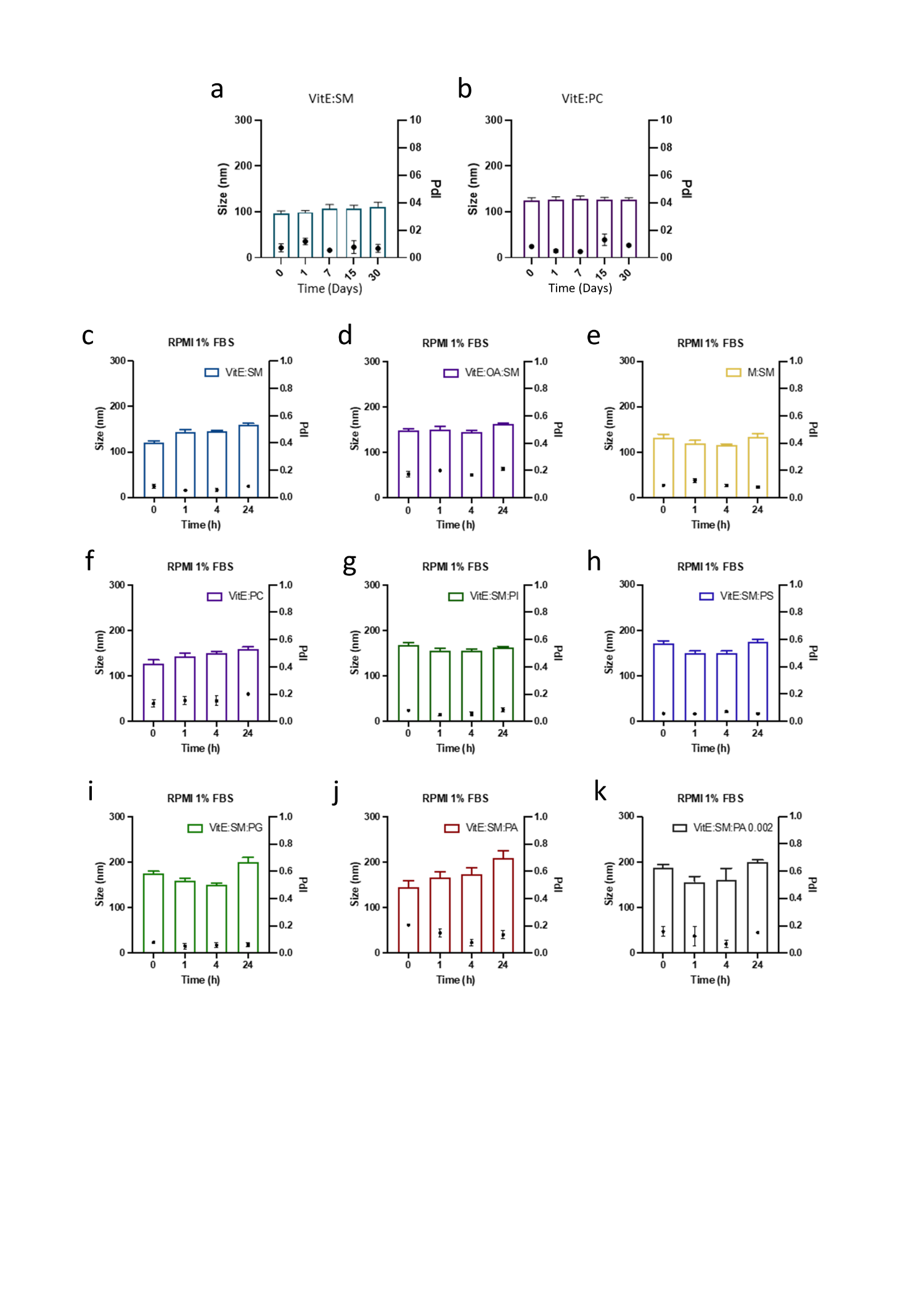


**Figure S1. Stability studies of the different lipid nanosystems. a-b)** Stability studies of the lipid nanosystems upon storage at 4°C over time (30 days): **a)** Vitamin E:Sphingomyelin, **b)** Vitamin E: Phosphatidylcholine. **c-k)** Stability studies of the lipid nanosystems determined at a 1:10 (v/v) dilution in a biorelevant media (i.e., 1% FBS supplemented RPMI): **c)** Vitamin E:Sphingomyelin, **d)** Vitamin E:Oleic acid:Sphingomyelin, **e)** Mygliol:Sphingomyelin, **f)** Vitamin E :Phosphatidylcholine, **g)** Vitamin E:Sphingomyelin:Phosphatidylinositol, **h)** Vitamin E:Sphingomyelin:Phosphatidylserine, **i)** Vitamin E:Sphingomyelin:Phosphatidylglycerol, **j)** and **k)** Vitamin E:Sphingomyelin:Palmitic acid at different concentrations of palmitic acid (see also Table 1). All measurements were taken under the same conditions; incubation at 37°C with orbital shaking (300 rpm) for up to 24h. Bar graph represent size ± SD and the scatter plot the average uniformity of a particle solution (PDI).


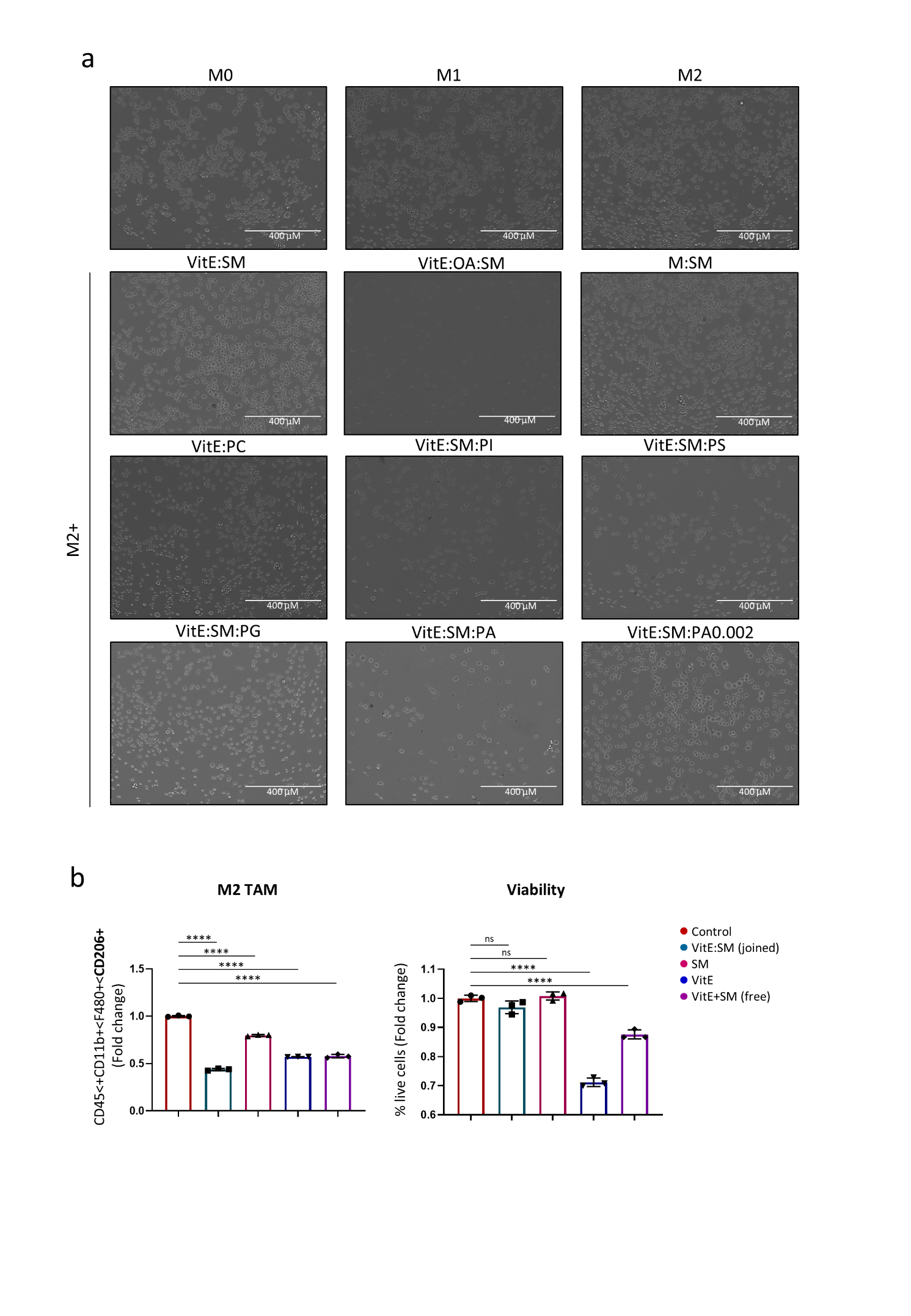


**Figure S2. Toxicity evaluation of different nanoemulsion compositions. a)** Representative light microscopy images of murine Immortalized Bone Marrow-Derived Macrophages (IBMDM) polarized to M1 using LPS + IFN-γ or M2 with IL4 in the presence or absence of different nanoparticle compositions (1 mg/mL): Vitamin E:Sphingomyelin, Vitamin E:Oleic acid:Sphingomyelin, Mygliol:Sphingomyelin, Vitamin E: Phosphatidylcholine, Vitamin E:Sphingomyelin:Phosphatidylinositol, Vitamin E:Sphingomyelin:Phosphatidylserine, Vitamin E:Sphingomyelin:Phosphatidylglycerol, and Vitamin E:Sphingomyelin:Palmitic acid at different concentrations of palmitic acid (see Figure 2b). Scale bar: 400µM. **b)** Analysis of the levels of the M2 marker CD45<+CD11b+<F480+<**CD206+** and toxicity (% live cells) as determined by flow cytometry in M2-polarized macrophages treated for 4h with VitE:SM nanoemulsions (1 mg/mL), SM (0.1 mg/mL), VitE (0.9 mg/mL), or a free combination of VitE and SM (0.9 mg/mL and 0.1 mg/mL). Bars represent the mean fold change ± SD (n = 3) in the indicted parameters, with untreated M2 (Control) set as 1.0. ∗ = p < 0.05; ∗∗ = p < 0.01; ∗∗∗ = p < 0.001; ∗∗∗∗ = p < 0.0001; ns=not significant. One-way ANOVA test for multiple comparisons with Dunnett’s post hoc test, compared to the untreated M2 (Control) sample.

**
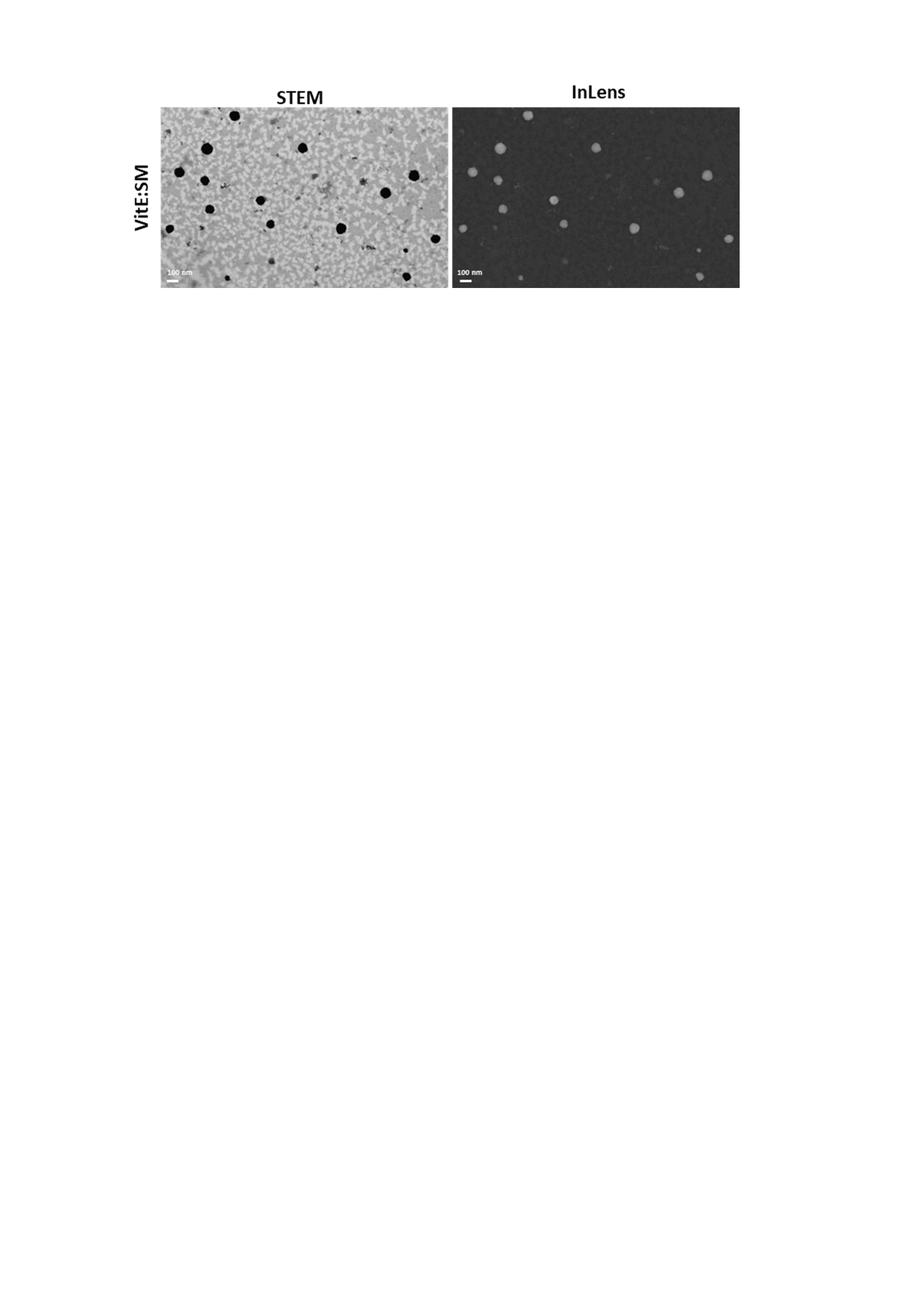
**

**Figure S3. Visualization of VitE:SM nanoemulsions.** FESEM images of VitE:SM nanosystems captured using two detector modes, STEM and InLens; Scale bar=100 nm.


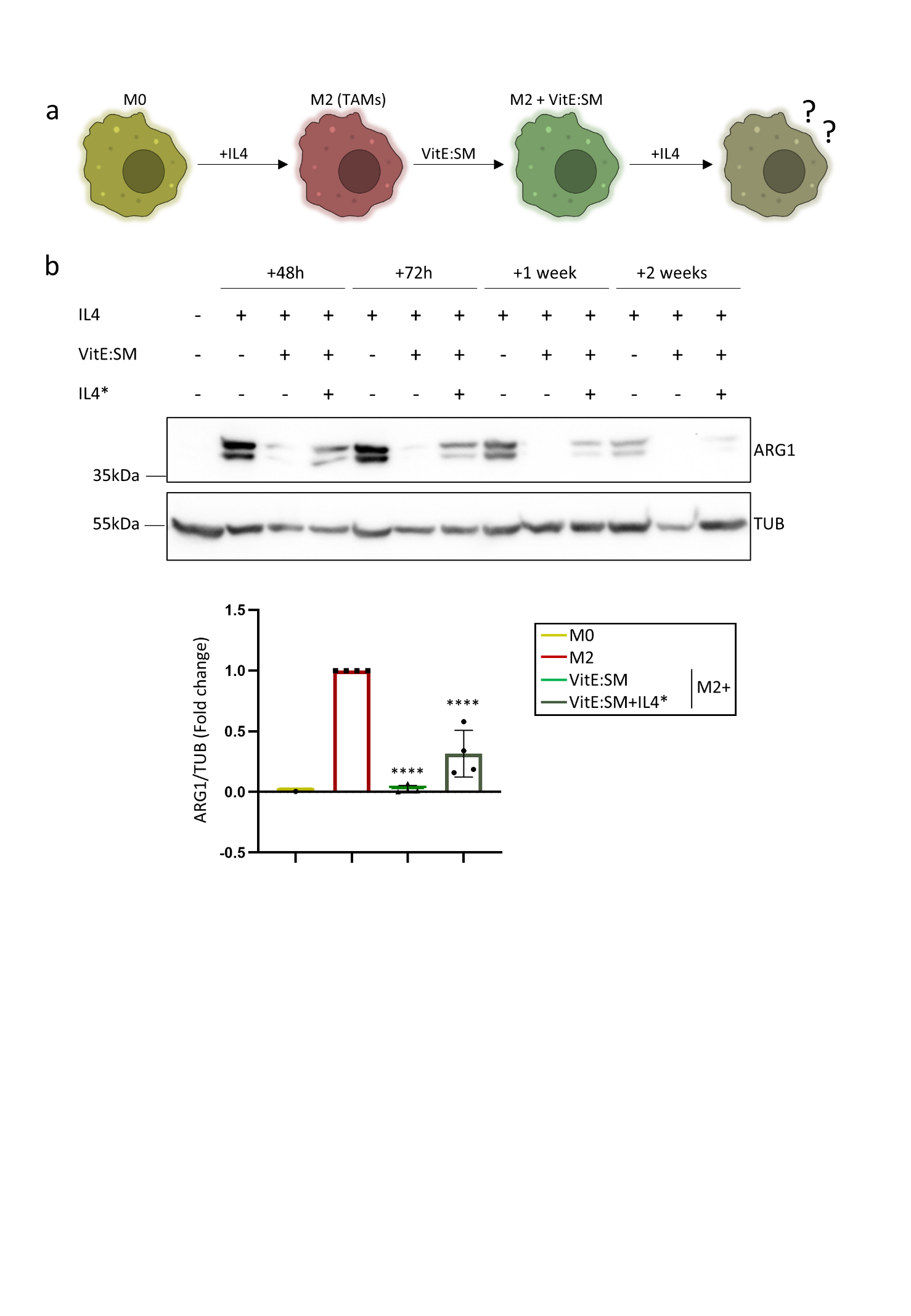


**Figure S4. VitE:SM lipid nanosystems-treated primary macrophages do not revert back to M2 TAMs despite re-stimulation. a)** Schematic illustrating the experimental assessment of the irreversibility of VitE:SM re-programming, even after a subsequent round of IL4 treatment (IL4*). **b)** Western immunoblot analysis depicting the levels of ARG1 protein over time in murine Bone Marrow-derived Macrophages (BMDM) polarized to M2 with IL4 and treated with VitE:SM nanoemulsions (0.5mg/mL). IL4* denotes a second round of polarization with IL4 following VitE:SM treatment. Densitometric analysis of the immunoblots is shown below, with the bars representing the mean fold change ± SD. (n = 4). ∗∗∗∗ = p < 0.0001. One-way ANOVA test for multiple comparisons with Dunnett’s post hoc test, compared to M2, set as 1.0.

**
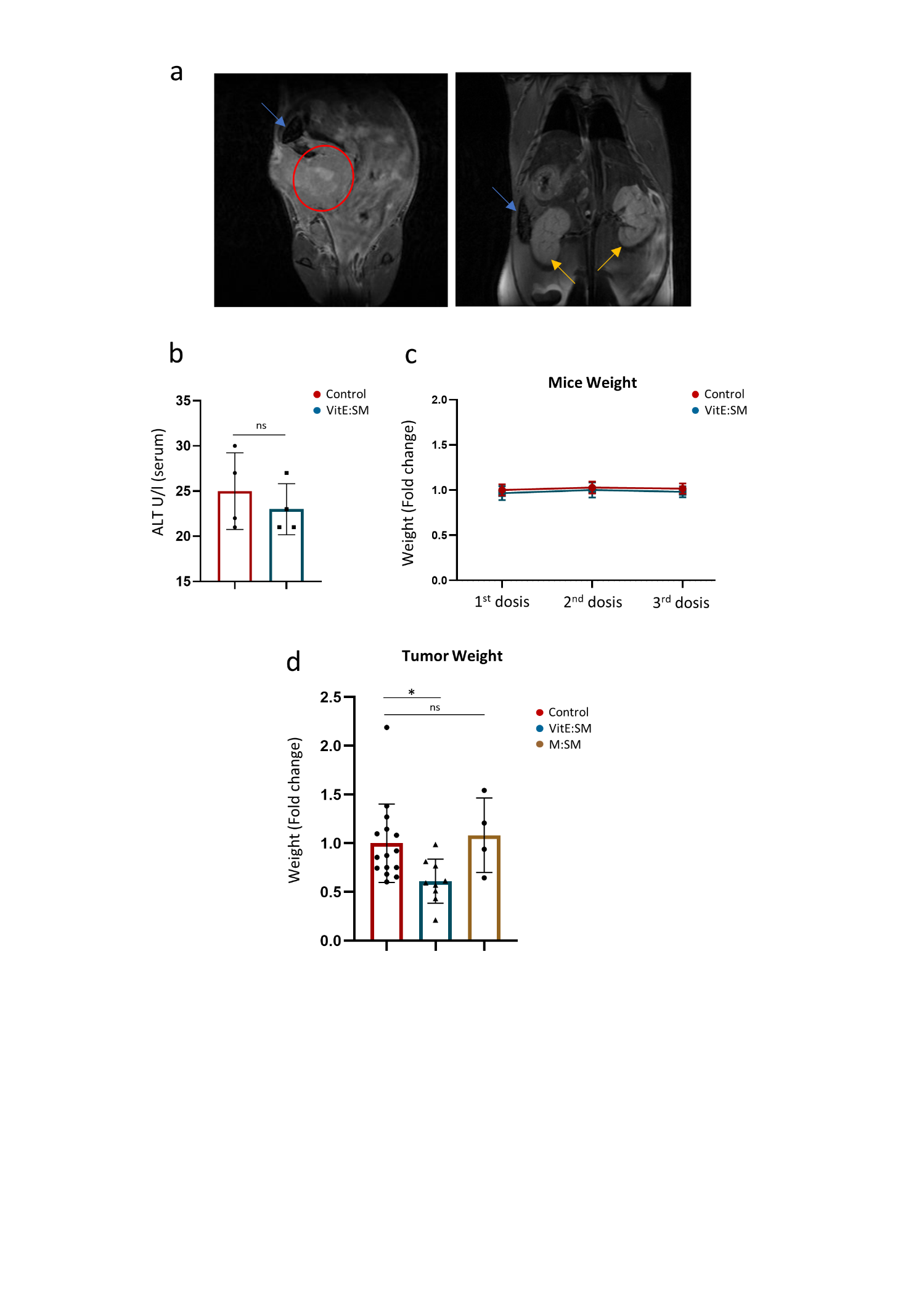
**

**Figure S5. Intraperitoneal injection of VitE:SM nanoemulsions reduces tumor burden in an orthotopic KPC tumor model. a)** Validation of orthotopic tumor pancreas development by MRI. Mice were scanned 3 weeks after tumor cell orthotopic injection. Red circle indicates the tumor inside the abdomen, blue arrows point to the head of the spleen and the yellow arrows indicate the kidneys. **b)** Evaluation of liver toxicity after VitE:SM nanoemulsion treatment: Liver function tests were performed on four representative mice treated with VitE:SM nanoemulsions (25mg/kg), receiving three doses every two days via retro-orbital injection over a one-week period. Serum analysis was conducted after treatment and compared to normal reference values: 28-129 U/L for ALT (alanine transaminase). **c)** Fold change ± SD in the total weight of the animals from b), measured at the start and daily throughout the treatment. Initial weights for both the control and VitE:SM groups (n=4) were pooled and normalized to 1.0 to track weight changes during the experiment. **d)** Fold change in tumor weight ± SD without the adjacent peritoneal tumor-derived masses from Figure 4g. Data were normalized for each duplicate experiment with the control set as 1.0. One-way ANOVA with Dunnett’s post hoc test, compared to control. ∗ = p < 0.05. ns=not significant.

**
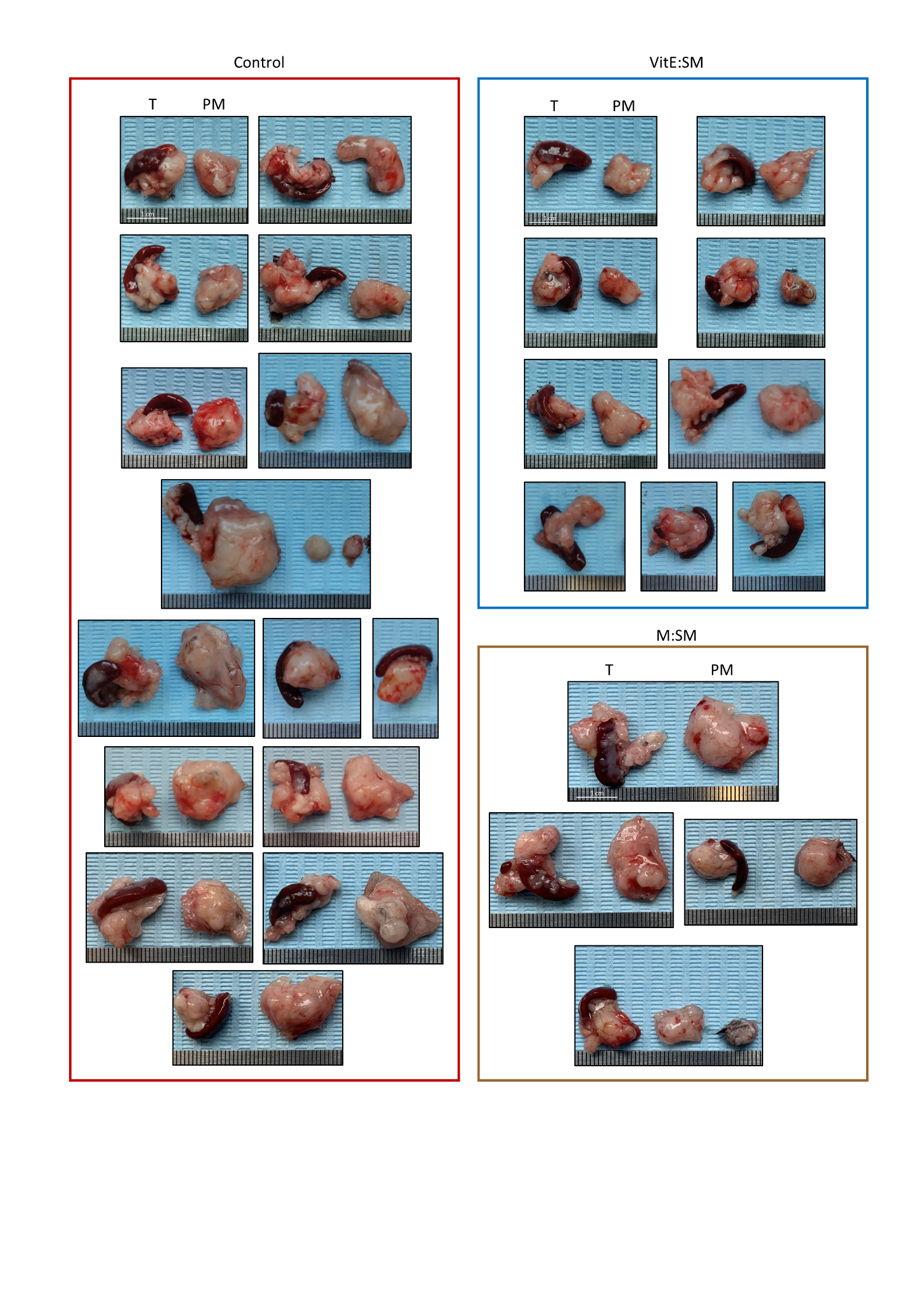
**

**Figure S6.** **Intraperitoneal injection of VitE:SM nanoemulsions reduces tumor burden in an orthotopic KPC tumor model.** All images from Figure 4g of the tumor (T) and adjacent peritoneum tumor-derived masses (PM) at the experimental endpoint for control (n=10) and VitE:SM-treated (n=9) groups from two independent experiments. An additional control group treated with non-effective nanoparticles M:SM (n=4) was included. Scale=1cm.


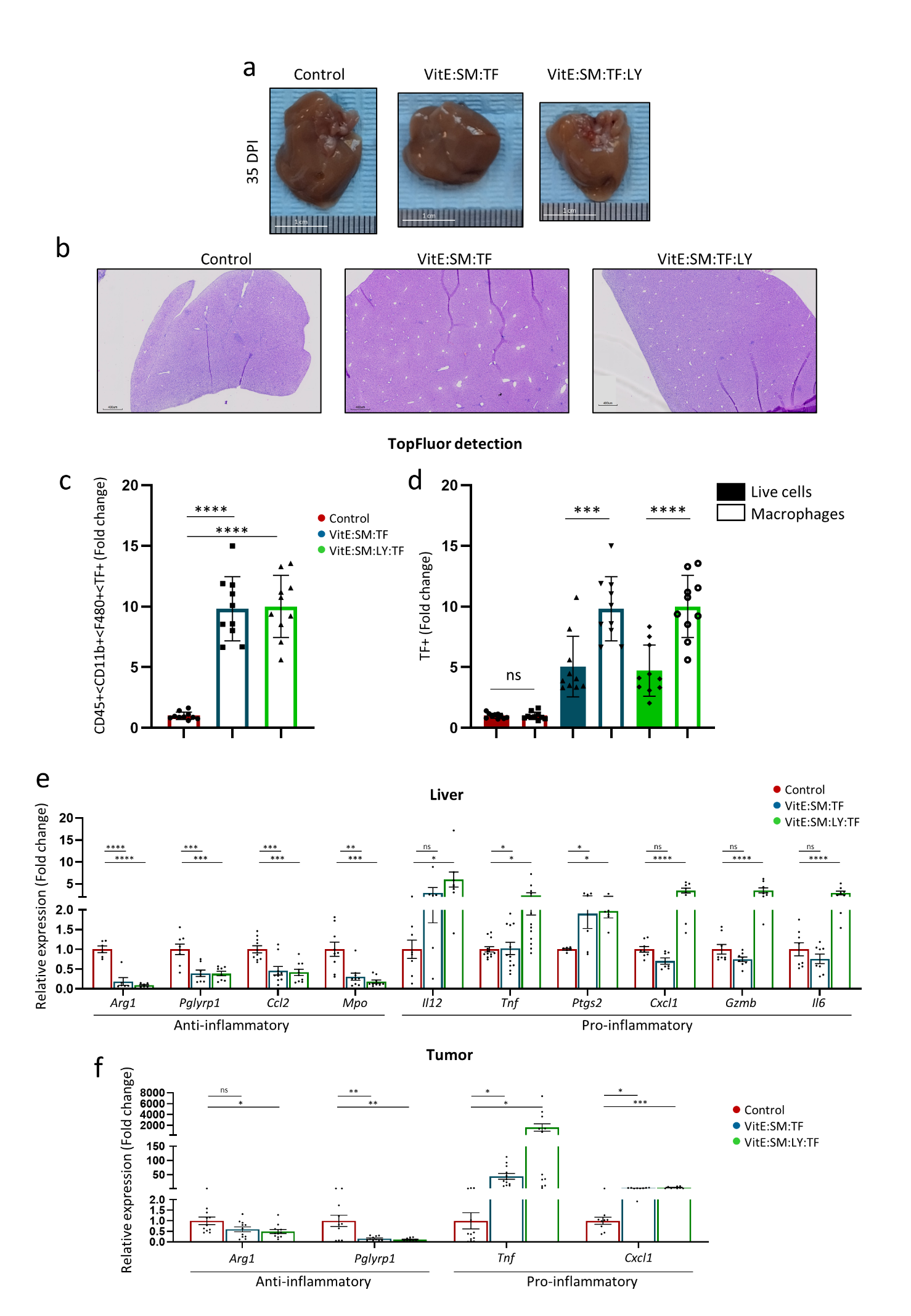


**Figure S7. TGF-βR1 inhibitor (LY2157299)-loaded VitE:SM nanoemulsions reduce tumor growth and diminishes TAM liver infiltration. a)** Representative macroscopic images of livers from the three experimental groups (Control, VitE:SM:TF and VitE:SM:LY:TF) at 35 days post-implant (DPI). Scale=1cm. **b)** Images (40X) of H&E-stained liver sections from control, VitE:SM:TF or VitE:SM:LY:TF-treated mice the 35 DPI mice. Scale=400µm. **c)** Flow cytometry analysis of TopFluor® signal in CD45+<CD11b+<F4/80+ liver macrophages for all experimental groups. One-way ANOVA test for multiple comparisons with Dunnett’s post hoc test, compared to Control, set as 1.0. ∗∗∗∗ = p < 0.0001. **d)** Flow cytometry analysis of TopFluor® signal in live cells (filled bars) vs CD45+<CD11b+<F4/80+ liver macrophages (empty bars) for all experimental groups. Bars represent the mean fold change ± SD. (n = 10). Unpaired t-test between live cells and macrophages in the three conditions. Control live cells sample was set as 1.0. ∗∗∗ = p < 0.001, ∗∗∗∗ = p < 0.0001, ns= not significant. **e-f)** qRT-PCR analysis of proinflammatory and anti-inflammatory cytokine markers from RNA extracted from representative liver (e) and tumor (f) samples from Figure 7. Bars represent the mean fold change ± SD. One-way ANOVA test for multiple comparisons with Dunnett’s post hoc test, compared to Control, set as 1.0. ∗ = p < 0.05; ∗∗ = p < 0.01; ∗∗∗ = p < 0.001; ∗∗∗∗ = p < 0.0001; ns=not significant.

**
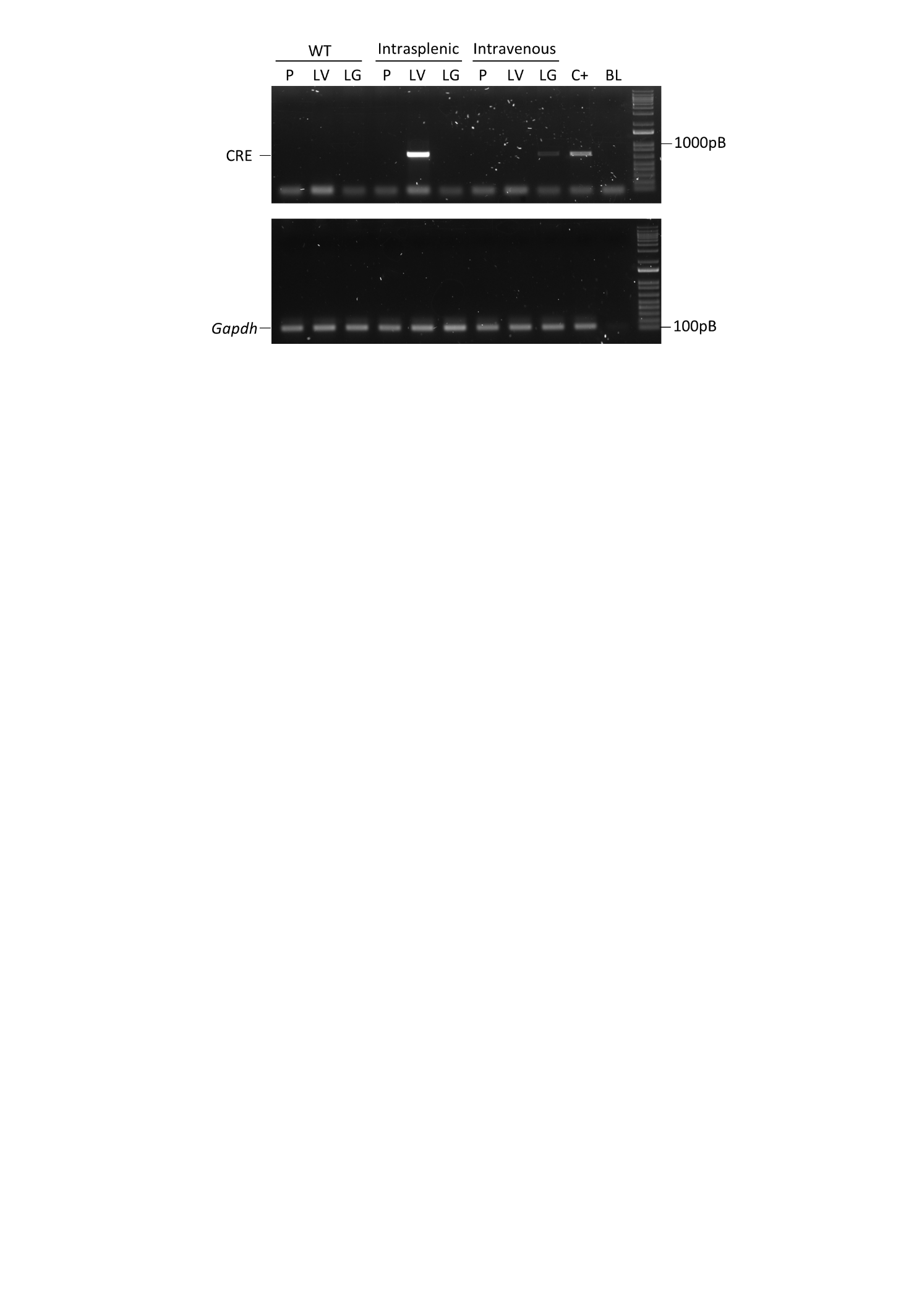
**

**Figure S8. Comparing intrasplenic and intravenous KPC cell injection models for liver metastasis formation.** PCR amplification of the gene CRE, expressed exclusively from KPC cells, in three different organs (P=Pancreas, LV=Liver and LG=Lung) in WT control mice or in the intrasplenic and intravenous model. Murine *Gapdh* was used as a housekeeping reference.

**
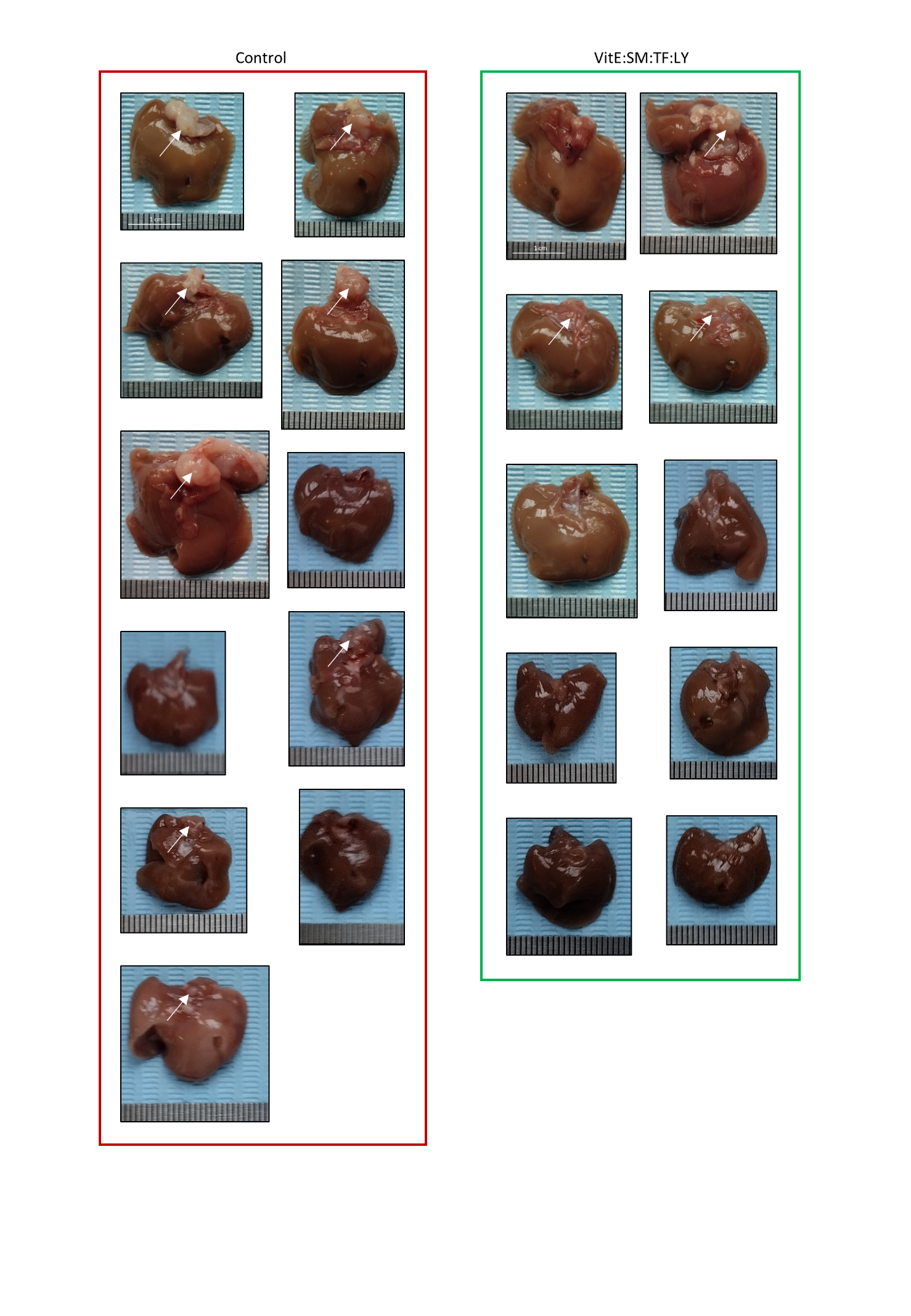
**

**Figure S9. TGF-βR1 inhibitor (LY2157299)-loaded VitE:SM nanoemulsions diminish liver metastasis in an intrasplenic KPC metastasis model.** All liver images from Figure 8d. Scale=1cm. White arrows point to macroscopic liver metastases.

**
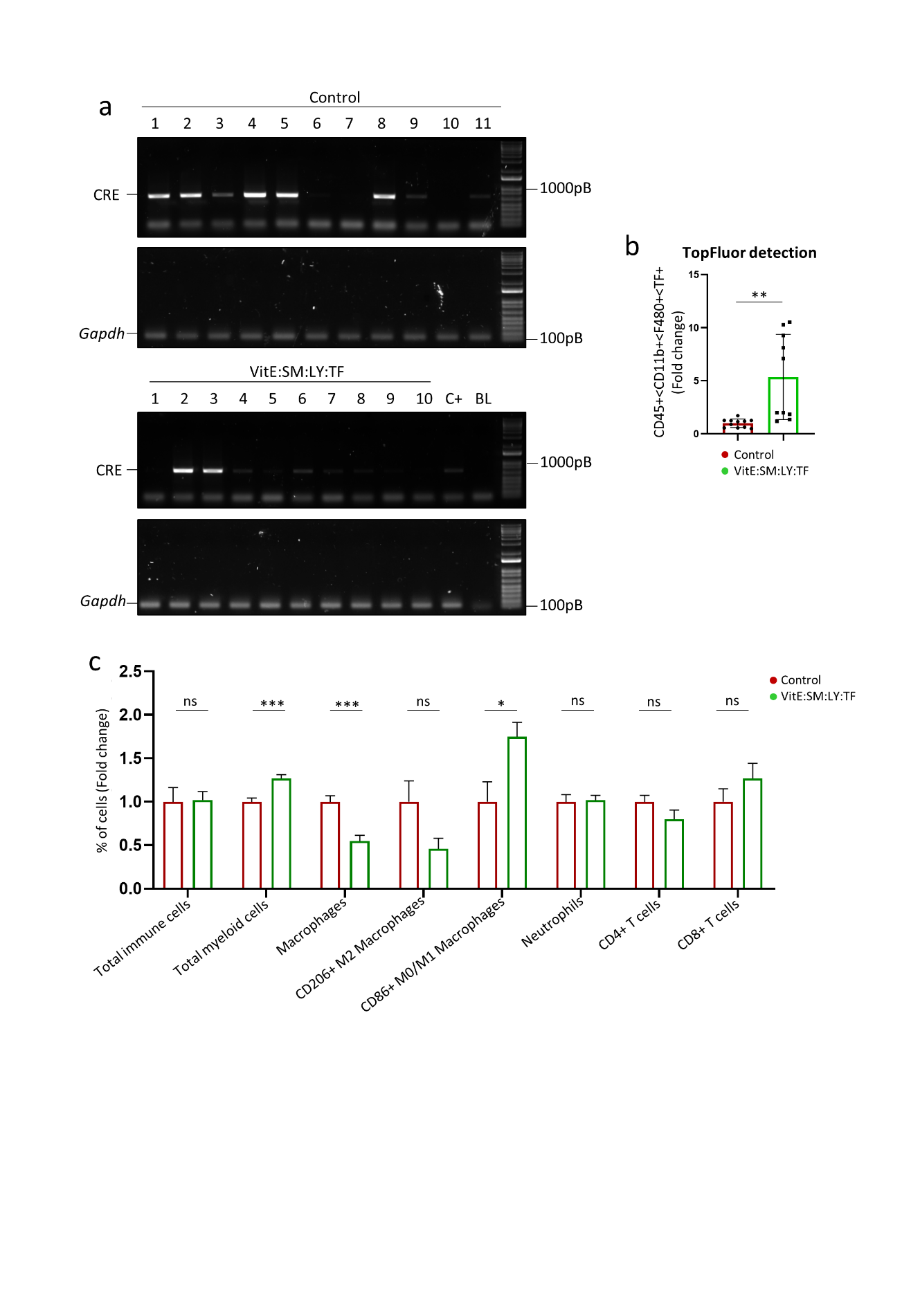
**

**Figure S10. TGF-βR1 inhibitor (LY2157299)-loaded VitE:SM nanoemulsions diminish liver metastasis in an intrasplenic KPC metastasis model. a)** PCR amplification of KPC cell-encoded CRE from DNA extracted from livers of Control or VitE:SM:LY:TF-treated mice injected intrasplenically with murine KPC cells. Murine *Gapdh* was used as housekeeping reference. **b)** Flow cytometry analysis of TopFluor expression in CD45+<CD11b+<F4/80+ liver macrophages. Bars represent the mean fold change ± SD (n = 10). **c)** Percentage of immune cell populations in liver samples extracted from representative Figure 8 FFPE paraffin blocks. Immune cell populations and flow cytometry gating: Total immune cells (CD45+), Myeloid cells (CD45+<CD11b+), Macrophages (CD45+<CD11b+<F4/80+), M2 macrophages (CD45+<CD11b+<F4/80+<CD206+), M1 macrophages (CD45+<CD11b+<F4/80+<CD86+), Neutrophils (CD45+<CD11b+<F4/80-<Ly6-G+), CD4+ T cells (CD45+<CD11b-<CD4+) and CD8+ T cells (CD45+<CD11b-<CD8+). Bars represent the mean fold change ± SD. Unpaired t-test to the Control sample, set as 1.0. ∗ = p < 0.05; ∗∗∗ = p < 0.001; ns=not significant.

**
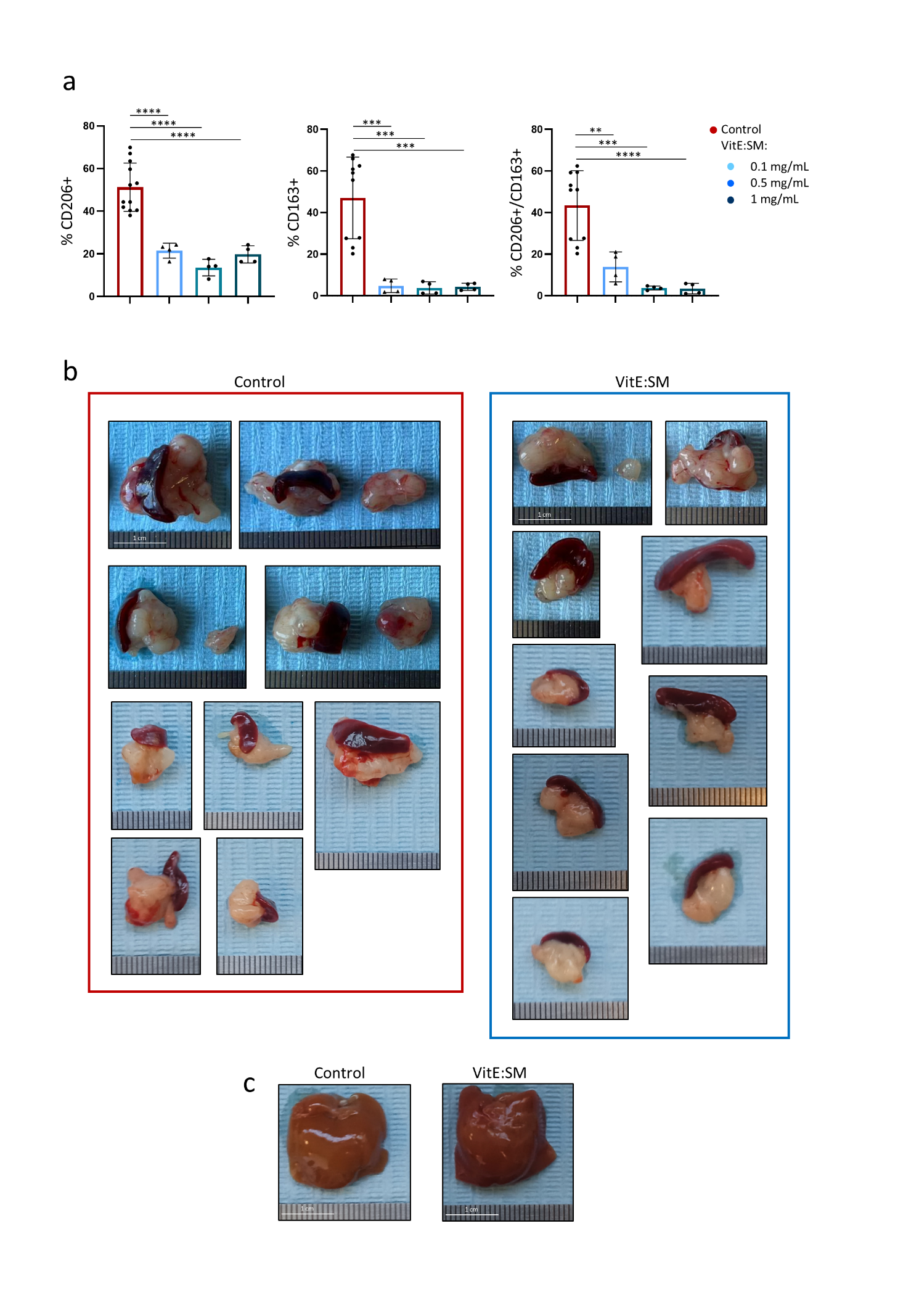
**

**Figure S11.** ***Ex vivo* treatment of human 3D tumor/CAF/macrophage spheroids with VitE:SM nanoemulsions. a)** Quantification, by flow cytometry, of the expression of CD206+, CD163+ or CD206+/CD163+ in human primary monocyte-derived M2-polarized macrophage cultures with VitE:SM nanoemulsions at the indicated concentrations (0.1, 0.5 or 1 mg/mL) for 4h. Bars represent the mean ± SD in the percentage of marker-positive cells. n=4-11, p values determined by One Way ANOVA test for multiple comparisons with Dunnett’s post hoc test, compared to Control. ∗∗ = p < 0.01; ∗∗∗ = p < 0.001; ∗∗∗∗ = p < 0.0001. **b)** All images from Figure 9d of the tumor (T) and adjacent peritoneum tumor-derived masses (PM) at the experimental endpoint for control (n=9) and VitE:SM-treated (n=9) groups from two independent experiments. Scale=1cm. **c)** Representative macroscopic images from livers from Figure 9d. Scale: 1cm.

**
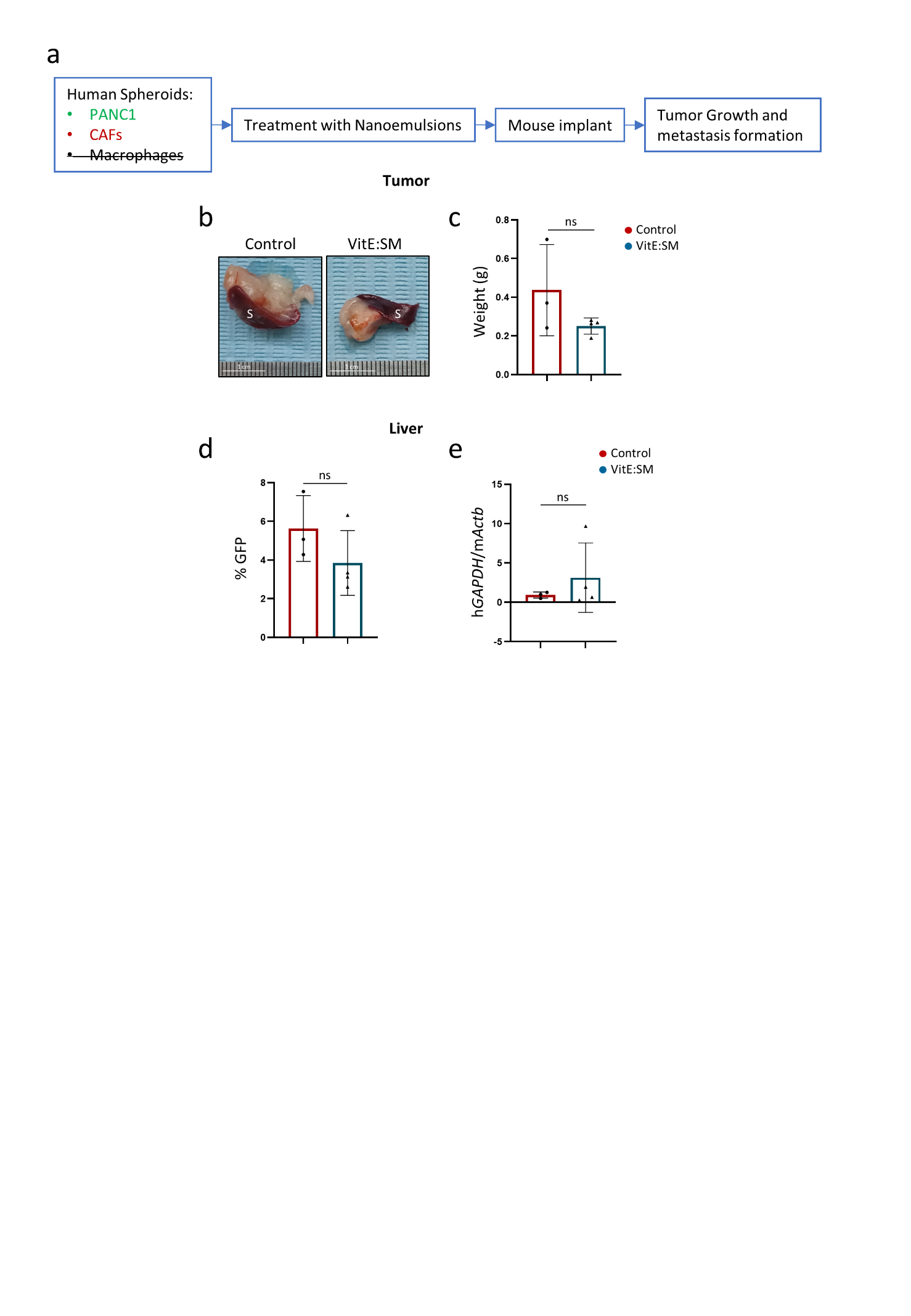
**

**Figure S12. *Ex vivo* treatment of human 3D tumor/CAF spheroids with VitE:SM nanoemulsions, excluding macrophages. a)** Schematic of human spheroid generation using pancreatic cancer cells (PANC1) and Cancer Associated Fibroblasts (CAFs), excluding macrophages. The experiment followed the same protocol as depicted in Figure 9a. **b)** Representative macroscopic images of tumors at the experimental endpoint (90 days post-implantation). The spleen (S) served as an anatomical reference for the pancreas. Scale=1cm. **c)** Histogram depicting tumor weights (g) ± SD from b). **d)** Percentage of GFP+ cells (PANC1) infiltrating the liver. Bars represent the mean ± SD. **e)** qRT-PCR analysis of the ratio of the human *hGAPDH* gene/murine beta-actin (*mActb*) gene. Bars represent the mean fold change ± SD, with Control set as 1.0. Data for Control (n=3) and VitE:SM (n=4) are presented. Unpaired t-test was applied, ns=not significant.
